# Hemp Seed-Based Foods and Processing By-Products Are Sustainable Rich Sources of Nutrients and Plant Metabolites Supporting Dietary Biodiversity, Health, and Nutritional Needs

**DOI:** 10.3390/foods14050875

**Published:** 2025-03-04

**Authors:** Ricardo Ramos-Sanchez, Nicholas J. Hayward, Donna Henderson, Gary J. Duncan, Wendy R. Russell, Sylvia H. Duncan, Madalina Neacsu

**Affiliations:** The Rowett Institute, University of Aberdeen, Foresterhill, Aberdeen AB25 2ZD, UK

**Keywords:** agricultural hemp, hemp seed-based foods, hemp-seed by-products, revalorization of by-products, dietary fibre, dietary protein, food biodiversity, circular nutrition, plant metabolites

## Abstract

Processing hemp seeds into foods generates several by-products that are rich in nutrients and bioactive phytochemicals. This paper presents a thorough plant metabolite analysis and a comprehensive assessment of the nutrient content of 14 hemp seed-based foods and by-products and evaluates their feasibility to deliver dietary needs and daily recommendations. The protein-85-product was the hemp food and hemp fudge the hemp by-product with the highest content of protein, 93.01 ± 0.18% and 37.66 ± 0.37%, respectively. Hemp seed-hull flour had the richest insoluble non-starch polysaccharide content (39.80 ± 0.07%). Linoleic acid was the most abundant fatty acid across all the hemp seed-based samples (ranging from 53.80 ± 2.02% in the protein-85-product to 69.53 ± 0.45% in the hemp cream). The omega-6 to omega-3 fatty acid ratio varied from 3:1 to 4:1 across all hemp seed-based samples. The majority of hemp seed-based samples were rich sources of potassium, magnesium, and phosphorus. Gentisic acid, p-coumaric acid, and syringaresinol were the most abundant plant metabolites measured and found mainly in bound form. Hemp seed by-products are valuable sources of nutrients capable of meeting dietary needs and, therefore, should be re-valorized into developing healthy food formulations to deliver a truly zero-waste hemp food production.

## 1. Introduction

Food waste represents a significant contributor to the release of greenhouse gases (GHGs) into the environment [1,2], calling for the implementation of highly efficient approaches that not only deliver comprehensive circular nutrition but also prioritize environmental sustainability [3]. In this regard, the search for food sources with minimal impact on climate change to supply and diversify essential dietary nutrients such as protein remains a research priority. Livestock production is recognized as one of the most common food sources to meet the need for protein in the global population [4]. Livestock production is accountable for at least of 12% of GHG emissions, being further compounded by a 30% decline in biodiversity resulting from human activities aimed at sustaining animal production [5,6]. This poses significant challenges to food security and sustainability. The limited availability of safe and nutritious food is one of the major concerns in food security, as this has been identified as a potential risk factor for the development of obesity, metabolic disorders, and cardiovascular diseases [7,8,9,10]. Solutions mitigating the negative impact on the environment and on human health caused by the intake of animal-based diets could be addressed by the increase in the consumption of sustainable plant-based food products [11,12].

Hemp seed is an important source of macronutrients, containing 29.34% of dietary protein [13], 33.02% of total fat [14], and 24.18% of insoluble fiber [15]. Moreover, hemp seeds are abundant in micronutrients [16], including potassium (690 mg/100 g–900 mg/100 g), phosphorus (410 mg/100 g–510 mg/100 g), and magnesium (420 mg/100 g–460 mg/100 g).

There is a limited amount of data available that compare the nutritional and non-nutrient phytochemical profile of hemp cake, hulls, and seed hearts derived from the food processing of whole hemp seeds. Hemp cake (hemp flour) is the main by-product obtained from pressing the seed for oil. Additionally, hemp contains rich amounts of nutrients and, therefore, could be harnessed by the food sector to develop novel food items and animal feed products. Hemp cake is an excellent source of micronutrients and macronutrients, as it provides between 28.53% and 32.06% protein, 32.21% and 34.14% fiber, and 9.02% fat [17,18]. Hemp by-products are rich in plant metabolites, some of which could replace the synthetic antioxidants currently used by the food industry for processes such as extending the shelf life of meat products [19,20,21,22]. The reduction in food waste and food processing waste could contribute to the reduction in GHG emissions, specifically in the production of CO_2_ [23]. Major contributors to the production of hemp cake are France with 81,485 metric tons, followed by Russia with 1376 metric tons, and China with 7684 metric tons [24]. Revalorizing food by-products could therefore bring additional economic gains and reduce the environmental impact of food production.

In this study, we characterized the macronutrient, micronutrient, and phytochemical content of hemp seed-based foods and by-products resulting from the cold pressing of hemp seed for oil and from the process of pasteurization of dehulled hemp seeds. This incorporates a range of samples, including whole seeds, hemp seed hearts, hemp oil, hemp cream, high protein products (protein-46-product, protein-75-product, and protein-85-product), hemp seed-hull flour, protein fiber boost, hemp cake, hemp fudge, and expellers. To our knowledge, the nutritional and non-nutrient phytochemical characterization of the by-product of cream solid residue (wet and dried), obtained from the process of milling and pasteurization of hemp seed hearts, is reported in this paper for the first time. The aim of this work is to understand the potential of hemp seed-based foods and by-products to diversify dietary nutrients and contribute towards meeting daily nutrient recommendations.

## 2. Materials and Methods

### 2.1. Standards and Reagents

The standards and reagents were mainly purchased from Sigma-Aldrich (Gillingham, UK) and Fisher Scientific UK Ltd. (Loughborough, UK) or synthesized based on protocols described previously [25,26]. ICP-MS analysis was performed using nitric acid of TraceSelect Ultra grade (Gillingham, UK), hydrochloric acid (30%) of Ultrapure grade (Merck; Darmstadt, Germany), and deionized water (Millipore; Bedford, MA, USA). Single-element standards were all purchased from Inorganic Ventures (Christiansburg, VS, USA).

### 2.2. Preparation of Plant Materials for Analyses

**Figure 1:**
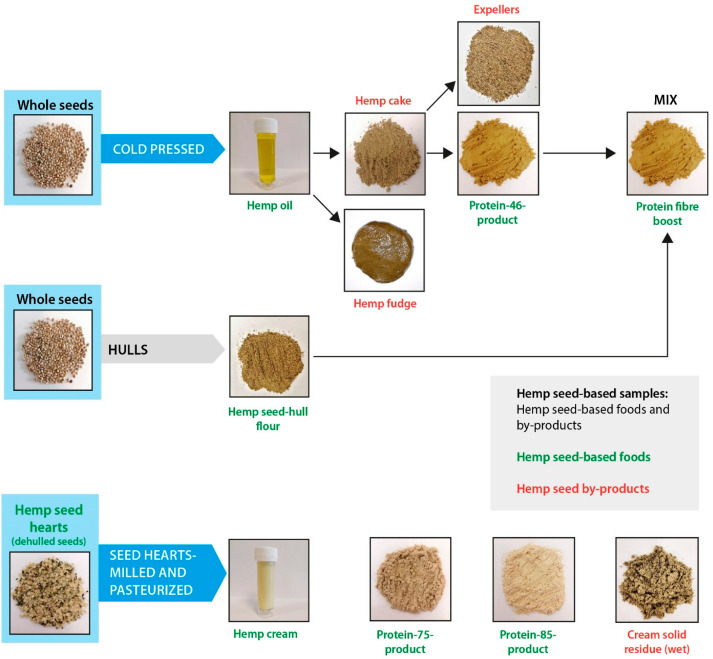
Flow diagram schematically describing all the hemp seed-based foods and by-products used for the analyses; where the hemp food products are depicted in green and the by-products in red color.

The samples analyzed in this study are those resulting from two different processes, namely cold pressing and milling–pasteurization (Figure 1). These processes deliver a range of hemp seed-based samples, including hemp seed-based foods and hemp by-products. In this regard, the hemp seed-based foods correspond to commercially available hemp foods, the hemp oil (obtained from cold pressing the seed), hemp cream (prepared from hemp hearts), protein fiber boost (obtained from mixing seed hull flour and protein-46 product), protein-75-product, protein-85-product (both obtained from hemp hearts, after de-hulling the seed), protein-46-product (obtained from concentrating the cake and extracting the fiber fraction (expeller)), and hemp seed-hull flour (obtained after seed de-hulling) (Figure 1). Following this, the hemp by-products include those obtained by pressing the seeds (hemp cake) and filtering the cold pressed oil (hemp fudge), and another is obtained after milling and pasteurizing the hemp seed hearts (cream solid residue (wet)), and after the production of protein 85- and 75-products, as shown in Figure 1. All the hemp seed-based samples were supplied by Braham & Murray Good Hemp (Barnstaple, UK). The protein-85-product is manufactured and commercialized with a minimum of 85% of protein. Hemp seed hearts, whole seeds, and hemp cake were freeze-milled (Spex 6700; Edison, NJ, USA). The cream solid residue (wet) was freeze-dried (Heto Lab Equipment; Allerød, Denmark) and then freeze-milled. The cream solid residue (dried) was spun at 1800× *g* (5 min, 5 °C) to eliminate most of the water, then freeze-dried to remove all the water and then freeze-milled to obtain a powder. As some molecules could be soluble in water, both the cream solid residue (wet) and cream solid residue (dried) were analyzed in terms of nutrients and plant metabolites. All the samples were vacuum-packed and stored cold at 4 °C prior to analysis. All hemp seed varieties used for industrial purposes must be grown under license from approved seed types with a tetrahydrocannabinol content not exceeding 0.2% [27].

### 2.3. Macronutrient Analysis

The macronutrient contents of the hemp seed-based foods and by-products were determined using proximate analysis. Protein was determined by the Dumas combustion method as the total nitrogen using a Vario Max CN analyzer (Elementar, Stockport, England) and by multiplying the preliminary results by 6.25 [28]. The resistant starch and soluble and insoluble non-starch polysaccharide contents were determined using previously published methods [29]. The total fat was determined by the Soxtec method using a Vario Max CN analyzer (Elementar; Stockport, UK) according to previous publications [30]. For the analysis of the fatty acid methyl esters (FAMEs), the fat was first extracted and analyzed by GC based on previously developed methods [31,32]. Samples (in the range of 50–200 mg) were suspended in methanol (2 mL) and macerated using an Ultra Turrax homogenizer (IKA T10, Staufen, Germany) at setting 4 (2800× *g*; 30 s at 5 °C). Then, chloroform (4 mL) was added, and the samples macerated again for a further 2 min and the supernatant separated and retained. The remaining precipitates were extracted with chloroform/methanol (5 mL, 2:1) and the supernatant was isolated and combined with the first supernatant obtained. Deionized water (6 mL) was added, and the samples were centrifuged at 1800× *g* (5 min, 5 °C) to separate the lower chloroform layer. Then, a gentle stream of nitrogen was further used to remove chloroform.

An aliquot of the extracted fat (20 mg) was mixed with hexane (0.5 mL) and methanolic HCl (methanol, 20 mL; acetyl chloride, 2 mL). Then, the samples were suspended in an internal standard of heptadecanoic acid (0.01 mL, 2 mg/mL in methanol). The samples were heated in a block heater at 105 °C for 2 h and, subsequently, taken out to allow them to cool down. Then, distilled water (2.5 mL) and hexane (2.5 mL) were added to the samples, and the top layers were separated by centrifugation at 1800× *g* (5 min, 5 °C). The samples were then extracted twice more with 2.5 mL of diethyl ether. The layers obtained were combined and mixed with distilled water (4 mL), and the lower aqueous layers produced were aspirated off. A volume of 5 mL distilled water was added to the samples, and the top layers were placed into stoppered tubes containing anhydrous sodium sulphate (1–2 cm). The samples were shaken well to separate any water from the solvents, and the solvents were evaporated under a stream of nitrogen (40 °C). The residues were mixed with butylated hydroxytoluene (0.1 mL; 0.02% in hexane) and then transferred to GC vials.

The separation of the compounds was performed by gas chromatography (Agilent Technologies 6890N, Stockport, England) equipped with a CP-SIL 88, 50 m × 0.25 mm column. The carrier gas was helium using a flow approximately 0.8 mL/minute at a pressure of 16 psi. The temperatures of the injector and detector were 250 °C and 270 °C, respectively. The oven temperature, registering 80 °C initially, was raised to 160 °C for 3 min, then further increased to 190 °C, with a final temperature of 230 °C that was maintained for 18 min.

### 2.4. Micronutrient Analysis

For the quantification of the microelement content, we used a method published previously [33]. Samples were suspended in nitric acid (8 mL; 65% (*v*/*v*) and distilled water (1 mL) using acid digest tubes and digested by a microwave-assisted digestion (MARS 6, CEM; Matthews, CA, USA). Two different temperature gradients were used to heat samples: (1) from 20 °C to 150 °C over 15 min and (2) 150 °C ramp to 165 °C over 10 min and then maintained at this temperature for 20 min. The measured isotopes assessed by ICP-MS were ^23^Na, ^24^Mg, ^31^P, ^39^K, ^44^Ca, ^51^V, ^52^Cr, ^55^Mn, ^56^Fe, ^59^Co, ^60^Ni, ^63^Cu, ^66^Zn, ^78^Se, ^95^Mo, ^111^Cd, ^202^Hg, and ^208^Pb. Nitric acid (2% *v*/*v*) and hydrochloric acid (0.5% *v*/*v*) were used as the decomposition matrix for making up all the solutions in distilled deionized water (Millipore, Bedford, MA, USA). The quantification of the microelement content was performed by ICP-MS using Agilent 7700X spectrometer (Agilent Technologies UK; Cheadle, UK) equipped with a MicroMist nebulizer and nickel sampler and skimmer cones. Erbium was the internal standard employed during this study (1 mg/L). The analysis had a duration of 3 min using a flow of 500 μL. Data acquisition was one point, five replicates, and 100 sweeps per replicate.

### 2.5. Extraction of Phytochemicals from Powders of Hemp Seed-Based Foods and Hemp By-Products

The method used for the extraction of plant metabolites was as described previously with minor modifications [34]. The free fraction was obtained when samples (0.1 g dry weight; *n* = 3) were suspended in hydrochloric acid (0.2 M; 3 mL) and extracted three times into ethyl acetate (5 mL). The organic layers were combined and filtered through sodium sulphate (anhydrous) and evaporated to dryness under reduced pressure at a temperature not exceeding 40 °C. The samples were reconstituted in LC-MS-grade methanol (0.5 mL) and stored at −70 °C.

The remainder of the aqueous fraction was neutralized to pH 7 with sodium hydroxide (4 M); then, ethyl acetate was removed from the samples (40 °C) under a stream of nitrogen and the samples were freeze-dried for 24–30 h. The pellets obtained were suspended in sodium hydroxide (1 M; 3 mL) and stirred for 4 h at room temperature. The pH was then reduced to pH 2 using hydrochloric acid (10 M), and the samples extracted with ethyl acetate; then, samples were evaporated to dryness and reconstituted in LC-MS-grade methanol (0.5 mL) and stored at −70 °C (alkali-labile fraction). The pH of the last aqueous fraction was raised to pH 7 with sodium hydroxide (4 M), and subsequently, the samples were subjected to a nitrogen stream and then freeze-dried. The acid-labile fraction was obtained by incubating the freeze-dried aqueous samples at 95 °C for 30 min with hydrochloric acid (2 M; 3 mL). Following this, the samples were extracted with ethyl acetate, evaporated to dryness, and reconstituted in 0.5 mL of LC-MS-grade methanol and stored at −70 °C. The thawed extracts were combined with internal standard 1 for negative-mode mass spectrometry (IS1; ^13^C benzoic acid; 2 mg/mL in 0.02% acetic acid in 80% methanol/water mixture; 50 μL) and internal standard 2 for positive-mode mass spectrometry (IS2; 2-amino-3,4,7,8-tetramethylimidazo[4,5-f] quinoxaline; 0.5 mg mL^−1^ in 0.02% acetic acid in 80% methanol/water mixture; 50 μL).

### 2.6. Extraction of Phytochemicals from Hemp Cream Product

The protocol used for the extraction of phytochemicals from the hemp cream sample was based on previous studies with some modifications [33,34]. Hemp cream (5 g; *n* = 3) was extracted three times into 5 mL of ethyl acetate, and the organic layers were separated by centrifugation at 1800× *g* (5 min, 5 °C) and then combined. The pellet obtained was extracted three times with LC-MS methanol (5 mL). The ethyl acetate and methanolic layers generated were combined and the solvent removed under reduced pressure at a temperature not exceeding 40 °C. The samples were reconstituted in LC-MS methanol (1 mL). The reconstituted sample (0.5 mL) was completely dried under nitrogen to prevent oxidation and submitted to hydrolysis for 1 h in 4 mL of 1 M HCl at 90 °C. Then, samples were extracted three times into 5 mL of ethyl acetate, and the layers were combined and dried under reduced pressure (40 °C). Samples were suspended in 0.5 mL LC-MS methanol and an aliquot of 0.1 mL was combined with internal standard 1 for negative-mode mass spectrometry (IS1; ^13^C benzoic acid; 2 mg/mL in 0.02% acetic acid in 80% methanol/water mixture; 50 μL) and internal standard 2 for positive-mode mass spectrometry (IS2; 2-amino-3,4,7,8-tetramethylimidazo[4,5-f] quinoxaline; 0.5 mg mL^−1^ in 0.02% acetic acid in 80% methanol/water mixture; 50 μL).

### 2.7. Extraction of Phytochemicals from Hemp Oil Product

The method used for the extraction of phytochemicals in hemp oil was based on a previous publication with minor modifications [35]. Hemp oil (5 g) was suspended in n-hexane (3 mL) and 6 mL of methanol/water (60:40 *v*/*v*). The methanol layer was removed and the extraction was repeated twice more, after which the methanolic layers were combined. Samples were washed three times with n-hexane (2 mL). The methanolic solution obtained was evaporated to dryness under reduced pressure at a temperature not exceeding 40 °C. Samples were reconstituted in methanol (1 mL) and an aliquot of 0.1 mL was combined with IS1 (negative mode) and IS2 (positive mode).

### 2.8. Plant Metabolite Analysis by LC-MS/MS

The separation of the plant metabolites, mainly benzoic acids, benzaldehydes, benzenes, acetophenones, cinnamic acids, phenylpropionic, phenylacetic, phenylpyruvic, phenyllactic acids, flavonoids, isoflavonoids, catechins, and lignans, was performed on an Agilent 1100 HPLC system (Agilent Technologies; Wokingham, UK) equipped with a Zorbax Eclipse 5 μm, 150 mm × 4 mm column (Agilent Technologies), using a previously published method [34]. Eluents obtained in the liquid chromatography were passed directly to an ABI 3200 triple quadrupole mass spectrometer (Applied Biosystems; Warrington, UK) fitted with a Turbo Ion Spray™ (TIS) source. The gradients were water with 0.1% acetic acid and acetonitrile containing 0.1% acetic acid. Method 1: 40–90% B (13 min), 90% B (1 min), 90–40% B (1 min), 40% B (9 min); method 2: 10–55% B (45 min), 55–80% B (15 min), 80% B (3 min), 80–10% B (0.2 min), 10% B (4.8 min); and method 3: 50–80% B (10 min), 80% B (2 min), 80–50% B (1 min), 50% B (4 min). The injection volume was 5 μL with a flow rate of 300 μL/min. All the compounds in the samples were assessed using multiple reaction monitoring and the standard solutions injected with a syringe pump. The molecular ion and the fragment ions were used to identify the ion transitions for each of the metabolites. In the case of those analytes that were not identified as their molecular and fragment ions were the same, the elution time was used to identify each of them. Furthermore, the voltage parameters, including declustering potential, collision energy, and cell entrance/exit potentials, were optimized individually for each analyte.

### 2.9. Statistical Analysis

All the samples were analyzed in triplicate, and the results are reported as mean ± standard deviation. Significant differences in macronutrients or phytochemicals were assessed by a one-way analysis of variance (ANOVA, (*p* < 0.05)) and Tukey’s test. Minitab Statistical Software (x64)—21.1.1.0 and Microsoft^®^ Excel^®^ for Office 365 (Microsoft Corporation, Redmond, Washington, DC, USA) were used for statistical analysis. The plant metabolites of the hemp samples were analyzed by principal component analysis (PCA), unit variance (UV)-scaled, using SIMCA 14.1 (Umetrics, Cambridge, UK).

## 3. Results and Discussion

### 3.1. Macronutrients

#### 3.1.1. Protein Content and Reference Nutrient Intakes Provided for the Hemp Seed-Based Samples

The protein, total fat, soluble non-starch polysaccharides (NSPs), insoluble NSPs, ash, and moisture contents are presented in Table 1. The protein-85-product, which is manufactured and commercialized with a minimum of 85% of protein, contained significantly higher (*p* < 0.01) amounts of protein (93.1%) compared to the figures of protein found in the rest of the hemp seed-based samples. The second highest concentration of protein was obtained for the protein-75-product (74.5%). The consumption of protein-75-product (67.6 g) and protein-85-product (54.3 g) can meet the recommended nutrient intake (RNI) of protein of 56 g/day and 45 g/day for men and women, which is 0.75 g protein per Kg body weight per day [36].

The content of protein among the by-products was significantly higher (*p* < 0.01) in the hemp fudge (37.6%), followed by the cream solid residue (dried) at 35.9%. One hundred grams of these by-products could contribute to meeting 75.3% and 71.9%, respectively, of the RNI for protein in adults. The amount of protein in hemp seed-hull flour (13.57%) and expellers (25.13%) along with seeds (26.67%) is shown in Table 1. These samples were similar to the values quantified in wheat (11%) and barley (20%) [37]. The content of protein measured in most of the hemp seed-based samples (Table 1) was found to be similar to those of soybean, lentil, chickpea, and pea (40%, 31%, 29%, and 31%, respectively) [38,39,40,41]. Additionally, the protein concentrations measured in hemp protein isolates are comparable with those reported other plant protein isolates, such as those from quinoa (73%), cowpea (76%), and lupin (91%). Moreover, the protein amounts obtained in 8 out of 14 hemp seed-based samples are similar to those from animal sources, including chicken breast (32%) and beef steak (31%) [36,42,43]. Food biodiversity is crucial for ensuring diversity in the human diet [44]. In this regard, the content of protein delivered by the hemp seed-based foods and by-products (Table 1) could complement traditional protein sources, such as meat-based sources (chicken and beef) [36], and other plant-based sources (favabean and green peas) [33]. Therefore, hemp seed-based samples potentially represent an opportunity to diversify the diet and deliver protein to promote healthier dietary patterns.

#### 3.1.2. Dietary Fiber

##### Comparison of Dietary Fiber Content of Hemp Seed-Based Samples with Other Sources of Dietary Fiber

The hemp seed-hull flour and expellers contained the highest concentrations of total soluble and insoluble NSPs, with 40.29% and 32.10%, respectively. These concentrations were comparable to a different work on commercially available defatted hemp flour (46.10%) and hemp cake (44.10%) obtained from seeds [45]. The total NSPs in protein fiber boost (19.93%) and hemp cake (24.65%) were also comparable to previous research on hemp flour (25.60%) and other plant proteins, for example, lupin (25.10%) [33]. The NSPs of the hemp seed-based samples are mainly found in insoluble form. The insoluble NSPs in hemp seed-hull fiber (39.80%) were found significantly higher (*p* < 0.01) compared to the rest of the hemp seed-based samples. The lowest concentration of NSPs was obtained in hemp seed hearts with 3.60% (Table 1). The insoluble NSP results obtained in hemp seed-hull fiber and hemp seed hearts (Table 1) were found in similar amounts to those of previous studies on hemp cake (39.90%) and durum wheat semolina (3.10%) [45,46]. The hemp seed-hull flour and expellers could therefore be used to contribute towards meeting the recommended dietary reference value [47] and to potentially diversify the sources of dietary fiber.

##### Effect of Food Processing on Dietary Fiber Composition of Hemp Seed-Based Samples and Their Potential Health Benefits

The monosaccharide composition of the soluble and insoluble NSPs is presented in Table 2. Xylose (23.90%) and glucose (9.27%) were the major components of the insoluble NSPs in the hemp seed-hull flour. Uronic acid along with arabinose (0.88% and 0.88% respectively) and glucose (1.20%) were the major components of insoluble NSPs in the protein-75-product. Arabinose (0.74%) and glucose (1.00%) were the major components of the insoluble NSPs in the hemp seed hearts. Xylose (18.60%) and glucose (6.85%) were the major components of the insoluble NSPs in the expellers. Rhamnose (5.01%) and uronic acid (2.11%) were the major components of the insoluble NSPs in the cream solid residue (dried). Xylose (2.06%) and uronic acid (1.83%) were the major components of insoluble NSPs in the cream solid residue (wet).

The cream solid residue (dried) was richer in rhamnose, while the cream solid residue (wet) in xylose in their NSP composition. Despite both samples being derived from an industrial process involving pasteurization, the application of high temperatures did not appear to impact on the hydrolysis of dietary fiber from NSPs. These results suggest that the water content of these by-products may be the factor associated with the differences observed in the monosaccharide composition. Some monosaccharides may be more likely to be soluble in water, which may explain the differences in rhamnose and xylose contents between these two by-products.

Xylose was identified in all the hemp seed-based samples and particularly quantified in higher concentrations in 6 out of 14 samples (Table 2). Xylose is a characteristic molecule found in different types of hemicelluloses, including arabinoxylans, xyloglucans, xylans, and xylo-oligosaccharides [48,49,50,51]. Hemicellulose reaches the large intestine, where it can be fermented by beneficial bacteria [48,50,51,52]. This information indicates that, if xylose was part of hemicellulose molecules, then the possible presence of prebiotic molecules in hemp seed samples could be suggested. The NSPs are responsible for various physiological effects in the small and large intestine, such as providing water dispersibility and viscosity effect, also being fermented by beneficial gut bacteria into short-chain fatty acids [53], explaining the strong interest within the food industry to integrate dietary fiber into staple foods to promote a healthier diet [54].

#### 3.1.3. Total Fat Content and Fatty Acid Composition

##### Fatty Acid Composition and Recommended Dietary Allowance Provided by Hemp Seed-Based Samples

Linoleic acid was the fatty acid delivered in higher amounts in all the hemp seed-based samples (Table 3). Additionally, the % of linoleic acid from the total fat (Table 3), but not the absolute value, was comparable to the % found in the fat content of two-row barley, maize, proso millet, rye, soybean, and triticale [55,56,57]. α-linolenic acid was the second most abundant in 9 out of 14 hemp samples analyzed. In the remaining hemp seed-based samples, oleic acid was identified as the second most abundant fatty acid (Table 3).

The concentration of palmitic acid in the protein-85-product (10.41%) was significantly higher (*p* < 0.05) compared to 10 out of 14 hemp seed-based samples. Additionally, the palmitic acid concentration in protein-85-product (10.41%) was similar to that of hemp cake (9.09%), protein-46-product (9.07%), and hemp seed-hull flour (9.03%). The lowest concentration of palmitic acid was found in hemp oil, with 5.93% (Table 3). These results are similar to those different cereals and oilseeds, including foxtail (11.0%), six-row barley (7.70%), proso (11.50%), linseed (7.00%), peanut (8.30%), and sunflower (8.00%) [55,56,57]. The stearic acid concentration was significantly higher (*p* < 0.01) in protein-85-product (4.24%). Moreover, the stearic acid concentration in all the hemp seed-based samples (Table 3) was comparable to those of two-row barley, oats, maize, and sorghum [55,56,57].

The fatty acids measured in all the hemp seed-based samples were similar to those of other studies on hemp seeds, hemp oil, and hemp cake [24]. On the other hand, the amounts of palmitic acid and stearic acid in the protein-85-product were significantly higher (*p* < 0.05) than those of most of the other hemp seed-based samples (Table 3). Moreover, the amounts of oleic acid, linoleic acid, γ-linolenic acid, and α-linolenic acid in the hemp cream were significantly (*p* < 0.05) higher than those of most of the other samples (Table 3). The fatty acid profile differences in the samples analyzed could be due to the process for their preparation from hemp seed, as the protein-85-product and hemp cream were both produced through a milling-pasteurization technology (Figure 1). Additionally, compared to the rest of the hemp seed-based samples, the protein-85-product exhibited significantly (*p* < 0.05) lower amounts of total fat, while the hemp cream delivered substantial amounts of total fat (Table 1). Therefore, potentially the molecular interactions between total fat and other nutrients during manufacturing process employed (milling–pasteurization) may be potentially linked to the differences observed in the fatty acid composition and content among the hemp seed-based samples.

The impact of food manufacturing processes on fat qualitative and quantitative profiles was also observed in the fatty acid composition of other hemp by-products. In this regard, most of the by-products had a similar fatty acid composition (*p* > 0.05), even when the content of total fat and manufacturing processes among the different by-products were different. For instance, the content of total fat between expellers and hemp cake was similar (*p* > 0.05), but significantly lower (*p* < 0.05) than that of cream solid residue (wet and dried). In turn, expellers and hemp cake are derived from the cold pressing of hemp seeds, while cream solid residue (wet and dried) are produced from the milling–pasteurization process (Table 3 and Figure 1).

The hemp seed-based foods and by-products could potentially contribute to the delivery of the recommended dietary allowance (RDA) for fatty acids [58,59,60]. For example, one hundred grams of protein-85-product delivers the highest levels of palmitic and stearic acid contributing to 34.6% and 61.0% towards the RDA. On the other hand, hemp cream could contribute to 32.6% of RDA for gamma-linolenic acid (Table S3). One hundred grams of all the hemp seed-based samples meet the entire RDA for linoleic and alpha-linolenic acid (Table S3). 

##### Omega-6/Omega-3 Ratios of Hemp Seed-Based Samples and Their Potential Health Benefits

Different types of by-products, including expellers, hemp cake, and cream solid residue (wet and dried), along with protein-85-product, protein-46-product, and hemp seed-hull flour, exhibited a ratio of omega-6/omega-3 (ω-6/ω-3) fatty acids of 4:1. In contrast, the ω-6/ω-3 ratio in the hemp oil, hemp cream, protein fiber boost, protein-75-product, hemp seed hearts, seeds, and the by-product of hemp fudge was 3:1 (Table 3). The research indicates that a ω-6/ω-3 ratio of 4:1 is associated with a significant 70% decrease in the overall mortality rate among individuals with cardiovascular disease [61]. Similarly, ratios within the range of 2–3:1 have been linked to reduction in inflammation levels in individuals suffering from rheumatoid arthritis. The Western-type diet is generally composed of meals with higher ω-6/ω-3 ratios ranging from 15:1 to 16.7:1 [62,63,64,65], which is linked to various chronic conditions, including cancer, cardiovascular disease, inflammatory and autoimmune diseases, obesity, and diabetes [61,66]. Consequently, incorporating hemp seed-based foods with beneficial ω-6/ω-3 ratios of 3:1 to 4:1, into the diet could contribute to the prevention of the development of numerous chronic diseases.

### 3.2. Mineral Content and the Reference Nutrient Intakes Provided by Hemp Seed-Based Samples

Of the hemp seed-based samples, 10 out of 14 were significantly (*p* < 0.05) richer in potassium, magnesiumm and phosphorus compared to the rest of the micronutrients (Table 4). Protein-46-product contained significantly higher amounts of potassium (1431.00 ± 49.08 mg/100 g) compared to the figures of potassium found in the rest of the hemp seed-based samples.

Potassium was detected in similar amounts between the cream solid residue (wet) and the cream solid residue (dried). Additionally, potassium (1190.85 ± 5.04 mg/100 g) was significantly higher in the cream solid residue (wet) than in the other by-products. The concentrations of magnesium and phosphorus in the protein-75-product (1581.36 ± 5.74 mg/100 g and 3096.66 ± 115 mg/100 g, respectively) were significantly higher than those of the rest of the hemp seed-based samples (Table 4). The concentrations of magnesium (607.67 ± 58.92 mg/100 g and 562.20 ± 44.65 mg/100 g, respectively) in hemp fudge and cream solid residue (dried) were significantly higher than those of the other by-products.

Phosphorus in cream solid residue (wet), cream solid residue (dried), and hemp fudge (1213.96 ± 14.6 mg/100 g, 1292.29 ± 113 mg/100 g, and 1267.16 ± 135 mg/100 g, respectively) was significantly higher than that of the other by-products. The content of magnesium, copper, zinc, and manganese in the seeds (Table 4) was similar to that of hemp seeds, as reported previously [24].

These results indicate that the manufacturing processes of the hemp seeds into hemp seed-based samples do not affect the mineral content profile. This implies that factors including the type of raw material (whole seeds or hemp seed hearts) and the temperature used during line processing do not lead to substantial alterations in the micronutrient composition.

One hundred grams of almost all the hemp seed-based samples (except for hemp seed-hull flour, hemp oil, and hemp cream) can provide all reference nutrient intake (RNI) for magnesium and phosphorus (Table S4). Moreover, one hundred grams of 12 out of the 14 hemp seed-based samples analyzed met all the recommendations for RNI for manganese and iron (with the exception of hemp oil and hemp cream) (Table S4). One hundred grams of protein fiber boost, protein-46-product, expellers, hemp cake, hemp fudge, cream solid residue (wet), and cream solid residue (dried) provided all the RNI for copper. Additionally, one hundred grams of protein-75-product, protein-85-product, protein-46-product, cream solid residue (wet), and cream solid residue (dried) delivered all the RNI for zinc (Table S4).

Incorporating hemp seed-based foods and by-products to fortify and reformulate existing food products could therefore help to meet the RNI for micronutrients. This, in turn, could contribute to addressing the increasing level of hidden hunger in the UK and other nations where access to diets with the capacity to meet essential dietary demands is restricted (i.e., Niger, Kenya, India, Uruguay, Armenia, Tunisia, and Malaysia) [67,68,69].

Nevertheless, even though hemp seed samples are rich in minerals, their bioavailability to humans could be affected by the presence of antinutrients, such as phytates, which may lead to a reduction in their nutritional quality. Previous work showed that phytate concentrations (3 g/100 g) in hemp seeds compromised the absorption of iron, zinc, and phosphorus [70]. Specifically, the molar ratios of phytates:iron (30.91) and phytates:zinc (35.59) in hemp seeds were shown to be much higher than those of the recommendations [70]. In this regard, a phytate:iron molar ratio > 1 is linked to a reduction in iron absorption [71], while a phytate/zinc molar ratio of 15 is linked to a reduction in zinc absorption [71]. On the other hand, the hemp seeds in that same study [70] showed potential for calcium absorption. Nevertheless, further studies are still necessary to evaluate micronutrient bioavailability from the hemp seed-based samples, specifically in vivo human dietary intervention studies. Moreover, food formulation work to reduce the phytates and increase mineral bioavailability is also necessary.

### 3.3. Phytochemical Composition

#### 3.3.1. Free and Bound Forms of Phytochemicals from Hemp Seed-Based Samples

**Figure 2:**
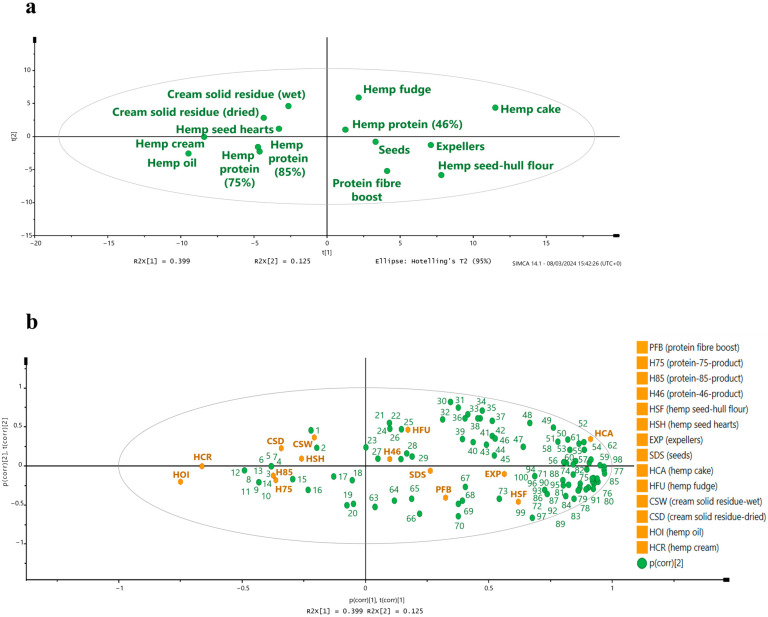
Principal component analysis (UV-scaled) of the plant metabolites analyzed by targeted LC-MS/MS analysis in all the hemp seed-based samples (**a**) and biplot showing the correlation between plant metabolites and hemp seed-based samples (**b**). Mandelic acid, (**1**); 3,4-dimethoxybenzaldehyde, (**2**); ferulic dimer (5–5 linked), (**3**); hydroxytyrosol, (**4**); matairesinol, (**5**); coumarin, (**6**); 4-hydroxy-3-methoxyphenylacetic acid, (**7**); hesperidin, (**8**); poncirin, (**9**); phloridzin, (**10**); neohesperidin, (**11**); hesperitin, (**12**); didymin, (**13**); daidzein, (**14**); phenyllactic acid, (**15**); indoe-3-lactic acid, (**16**); caffeine, (**17**); glycitein, (**18**); coumesterol, (**19**); bergapten, (**20**); luteolin, (**21**); morin, (**22**); epicatechin, (**23**); benzoic acid, (**24**); 4; hydroxyphenyllactic acid, (**25**); 3,4-dihydroxyphenylpropionic acid, (**26**); quercetin, (**27**); 4-hydroxy-3-methoxyphenylpropionic acid, (**28**); indole-3-pyruvic acid, (**29**); indole-3-acetic acid, (**30**); ferulic acid, (**31**); sinapic acid, (**32**); kaempferol, (**33**); kynurenic acid, (**34**); gentisic acid, (**35**); 4-ethylphenol, (**36**); o-anisic acid, (**37**); m-coumaric acid, (**38**); scopoletin, (**39**); phenylpyruvic acid, (**40**); catechin, (**41**); naringenin, (**42**); isorhamnetin, (**43**); isoliquiritigenin, (**44**); ethylferulate, (**45**); niacin, (**46**); chlorogenic acid, (**47**); 2,3-dihydroxybenzoic acid, (**48**); 4-hydroxyphenylacetic acid, (**49**); apigenin, (**50**); 3,4-dihydroxymandelic acid, (**51**); vitexin, (**52**); tyrosol, (**53**); anthranilic acid, (**54**); cinnamic acid, (**55**); naringin, (**56**); genistein, (**57**); 3-hydroxymandelic acid, (**58**); vanillin, (**59**); quinadilic acid, (**60**); p-hydroxybenzoic acid, (**61**)**;** protocatachaldehyde, (**62**); 8-methylpsoralen, (**63**); coniferyl alcohol, (**64**); myricetin, (**65**); imperatorin, (**66**); quercitrin, (**67**); tangeretin, (**68**); luteolinidin, (**69**); formononetin, (**70**); rutin, (**71**); 4-hydroxyphenylpyruvic acid, (**72**); taxifolin, (**73**); 4-hydroxyacetophenone, (**74**); 4-hydroxy-3-methoxyacetophenone, (**75**); caffeic acid, (**76**); 2,6; dihydroxybenzoic acid, (**77**); phenylacetic acid, (**78**); indole, (**79**); quercetin-3-glucoside, (**80**); p-coumaric acid, (**81**); syringin, (**82**); syringic acid, (**83**); hyperoside, (**84**); 3-hydroxyphenylpropionic acid, (**85**); i3-carboxaldehyde, (**86**); indole-3-carboxylic acid, (**87**); ferulic dimer (8-5 linked), (**88**); 4-hydroxy-3,5-dimethoxyacetophenone, (**89**); protocatechuic acid, (**90**); vanillic acid, (**91**); salicylic acid, (**92**); syringaresinol, (**93**); phenol, (**94**); p-hydroxybenzaldehyde, (**95**); secoisolariciresinol, (**96**); 4-hydroxymandelic acid, (**97**); 4-methoxycinnamic acid, (**98**); 4-hydroxy-3-methoxymandelic acid, (**99**); pinoresinol, (**100**).

The principal component analysis (PCA) of the plant metabolites analyzed in the hemp seed-based foods and by-products showed that hemp oil, hemp cream, protein-85-product, and protein-75-product clustered in the lower left-hand quadrant, suggesting similar plant metabolite profiles (Figure 2a). This clustering is likely to be due to the similar amounts of phenyllactic acid, indole-3-lactic acid, coumesterol, glycitein, and 4-hydroxy-3-methoxyphenylacetic acid found in these samples (Figure 2b, and Tables S1 and S2). The clear segregation of seeds, protein fiber boost, expellers, and hemp seed-hull flour (Figure 2a) is likely to be due to the high levels of syringaresinol, p-coumaric acid, salicylic acid, and syringin present in these samples (Figure 2b, and Tables S1 and S2).

The PCA showed that protein-46-product, hemp fudge, and hemp cake were situated in the upper right-hand quadrant (Figure 2a). These samples contained high amounts of indole-3-pyruvic acid, gentisic acid, benzoic acid, m-coumaric acid, vanillin, and ferulic acid compared to the cluster formed by cream solid residue (wet), cream solid residue (dried), and hemp seed hearts (Figure 2b, and Tables S1 and S2). Therefore, the segregation between these two groups suggests different plant metabolite profiles.

**Figure 3:**
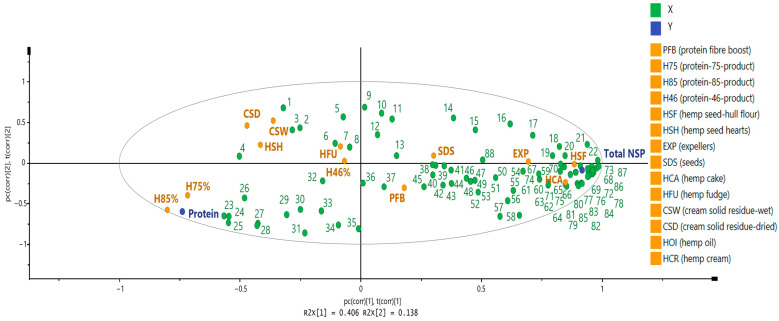
Partial least squares-discriminant analysis of the content of protein, total NSPs, and the plant metabolites analyzed by targeted LC-MS/MS analysis in all the hemp seed-based samples. Mandelic acid, (**1**); 3,4-dimethoxybenzaldehyde, (**2**); benzoic acid, (**3**); indole-3-pyruvic acid, (**4**); luteolin, (**5**); morin, (**6**); gentisic acid, (**7**); epicatechin, (**8**); kynurenic acid, (**9**); ferulic acid, (**10**); 3,4-dihydroxyphenylpropionic acid, (**11**); indole-3-acetic acid, (**12**); kaempferol, (**13**); sinapic acid, (**14**); 2,3-dihydroxybenzoic acid, (**15**); 4-hydroxyphenylacetic acid, (**16**); 3,4-dihydroxymandelic acid, (**17**); cinnamic acid, (**18**); anthranilic acid, (**19**); p-hydroxybenzoic acid, (**20**); tyrosol, (**21**); 4-hydroxy-3-methoxyacetophenone, (**22**); phenyllactic acid, (**23**); glycitein, (**24**); indoe-3-lactic acid, (**25**); 4-hydroxyphenyllactic acid, (**26**); coumesterol, (**27**); caffeine, (**28**); 4-hydroxy-3; methoxyphenylpropionic acid, (**29**); bergapten, (**30**); coniferyl alcohol, (**31**); quercetin, (**32**); 8-methylpsoralen, (**33**); imperatorin, (**34**); tangeretin, (**35**); phenylpyruvic acid, (**36**); myricetin, (**37**); 4-ethylphenol, (**38**); o-anisic acid, (**39**); chlorogenic acid, (**40**); m-coumaric acid, (**41**); catechin, (**42**); quercitrin, (**43**); niacin, (**44**); formononetin, (**45**); scopoletin, (**46**); naringenin, (**47**); isorhamnetin, (**48**); isoliquiritigenin, (**49**); ethylferulate, (**50**); apigenin, (**51**); taxifolin, (**52**); rutin, (**53**); vitexin, (**54**); quinadilic acid, (**55**); genistein, (**56**); 4-hydroxyphenylpyruvic acid, (**57**); phenylacetic acid, (**58**); protocatachaldehyde, (**59**); 3-hydroxyphenylpropionic acid, (**60**); 2,6-dihydroxybenzoic acid, (**61**); 4-hydroxymandelic acid, (**62**); phenol, (**63**); ferulic dimer (5-5 linked), (**64**); 4-methoxycinnamic acid, (**65**); p-hydroxybenzaldehyde, (**66**); 4-hydroxy-3-methoxymandelic acid, (**67**); naringin, (**68**); 3-hydroxymandelic acid, (**69**); quercetin-3-glucoside, (**70**); vanillin, (**71**); vanillic acid, (**72**); syringic acid, (**73**); syringin, (**74**); 4-hydroxy-3,5-dimethoxyacetophenone, (**75**); indole-3-carboxylic acid, (**76**); p-coumaric acid, (**77**); I3-carboxaldehyde, (**78**); syringaresinol, (**79**); 4-hydroxyacetophenone, (**80**); caffeic acid, (**81**); salicylic acid, (**82**); indole, (**83**); pinoresinol, (**84**); hyperoside, (**85**); protocatechuic acid, (**86**); secoisolariciresinol, (**87**); luteolinidin, (**88**).

The partial least squares-discriminant analysis (PLS-DA) highlighted several plant metabolites and hemp seed-based samples potentially associated with the content of protein. Phenyllactic acid, 4-hydroxyphenyllactic acid, 4-hydroxy-3-methoxyphenylpropionic acid, and coniferyl alcohol (Figure 3 and Table S1) were richer in samples with high amounts of protein (i.e., protein-46-product (55.6%), protein-75-product (74.5%), and protein-85-product (93.01%)). These plant metabolites were positively associated with the content of protein, as can be seen in the PLS-DA plot (in Figure 3). 

The PLS-DA showed that protein fiber boost, hemp seed-hull flour, expellers, and hemp cake clustered in the lower right-hand quadrant. These samples contained high amounts of total NSP (19.9% to 40.3%) and high levels of p-coumaric acid, syringaresinol, vanillin, syringin, and salicylic acid, which were found to be positively correlated with the amount of total NSP (Figure 3, and Tables S1 and S2).

**Figure 4:**
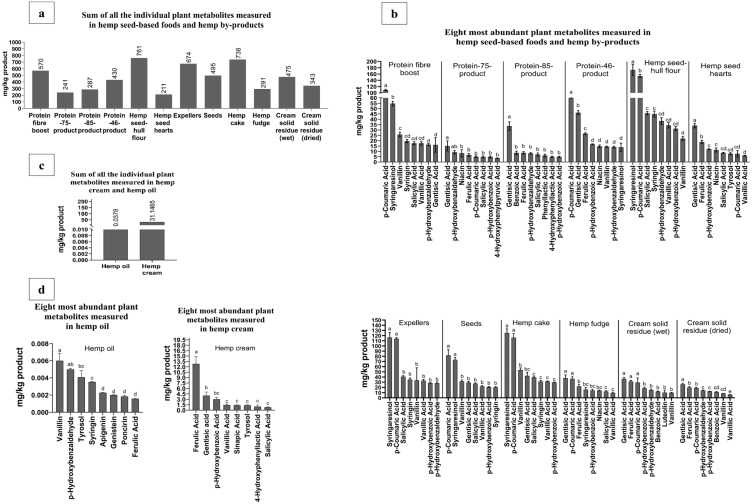
The total plant metabolite content in hemp powders (mg/kg dry product), obtained by summing the individual plant metabolites measured by LC-MS/MS, (**a**); the eight most abundant individual plant metabolites (mg/kg dry product) measured in hemp seed-based foods and by-products, (**b**); the total plant metabolite content of hemp oil and hemp cream (mg/kg) obtained by summing the individual plant metabolites measured by LC-MS/MS, (**c**); the eight most abundant individual plant metabolites (mg/kg) measured in hemp oil (**d**) and hemp cream. Data within each sample with different letters are significantly different (*p* < 0.05).

The amount of total plant metabolites (obtained by summing all the individual values of the plant metabolites from the hemp seed-based samples) was analyzed using targeted LC-MS/MS analysis. Hemp seed-hull flour (761 mg/kg), protein fiber boost (570 mg/kg), and protein-46-product (430 mg/kg) were the hemp seed-based foods with the highest content of plant metabolites. Among the by-products, hemp cake (738 mg/kg), expellers (674 mg/kg), and cream solid residue (wet) with 475 mg/kg contained the largest concentrations of plant metabolites measured (Figure 4a). The results from the present study were compared to those from previous studies [72,73]. In this regard, the sum of plant metabolites from each of the hemp seed-based samples was higher compared to that of green pea and yellow pea (129 mg/kg and 214 mg/kg) [72]. Moreover, the sum of plant metabolites from each of the hemp seed-based samples (Figure 4a) was lower than that of UK-produced hulled buckwheat (1159 mg/kg) [73]. In contrast, hemp oil (0.0378 mg/kg) and hemp cream (31.1 mg/kg) had the lowest amounts of plant metabolites (Figure 4c).

p-Coumaric acid was the most abundant plant metabolite (*p* < 0.01) found in protein fiber boost and protein-46-product. Moreover, p-Coumaric acid was the most abundant plant metabolite measured in seeds (Figure 4b). Gentisic acid was the most abundant plant metabolite (*p* < 0.05) found in protein-75-product, protein-85-product, hemp seed hearts, and cream solid residue (dried). Additionally, gentisic acid was the most abundant plant metabolite in hemp fudge and cream solid residue (wet). Both p-coumaric acid and gentisic acid (Figure 4b) were measured following alkaline hydrolysis (NaOH, 1M) and acid hydrolysis (HCl, 2M), and therefore were bound to distinct types of plant components.

Syringaresinol was the most abundant plant metabolite (*p* < 0.01) found in hemp seed hull-flour. Moreover, syringaresinol was the most abundant plant metabolite measured in expellers and hemp cake (Figure 4b and Tables S1 and S2). Specifically, syringaresinol in these three samples was found to be mainly in bound form and extracted only after alkaline and acid hydrolyses.

The rest of the other plant metabolites measured in the hemp seed-based samples analyzed are presented in Figure 4b, and Tables S1 and S2. On the other hand, in the hemp oil, vanillin was found as the most abundant plant metabolite, followed by p-hydroxybenzaldehyde and tyrosol (Figure 4d). In the hemp cream, the most abundant plant metabolite found in significant amounts (*p* < 0.01) was ferulic acid.

The second most abundant plant metabolite in hemp cream was gentisic acid, followed by p-hydroxybenzoic acid (Figure 4d). Hemp seed-based foods and their by-products were also rich in p-hydroxybenzoic acid (detectable in twelve samples), salicylic acid (detectable in ten samples), vanillin, p-hydroxybenzaldehyde and vanillic acid (detectable in nine samples), and syringin (quantified in only six samples).

The amounts of syringaresinol in expellers and hemp cake (Figure 4b) were identified to be twice as high as those found in the hemp seed screenings, and also released following alkaline and acid hydrolyses [74]. In contrast, the content of syringaresinol extracted after enzymatic hydrolysis on hemp seeds [75] was found to be twice as high as those found in the expellers and cake from this study (Figure 4b). The content of gentisic acid in the rest of the by-products from this work (Figure 4b) was detectable and higher than that of previously reported hemp screenings [74]. The concentration of p-coumaric acid in the protein-46-product and seeds (Figure 4b) was similar to that of a work on hemp cake (67.9 mg/kg), which employed the method of subcritical water extraction to obtain the plant metabolites [76]. The concentration of ferulic acid in the hemp cream (Figure 4d) was much higher than that of hemp oil (0.73 mg/kg) [77], which obtained ferulic acid from alkaline and acid hydrolyses.

p-Hydroxybenzaldehyde was the second most abundant plant metabolite in the protein-75-product (9.19 ± 1.63 mg/kg). Benzoic acid was the second most abundant plant metabolite in the protein-85-product (8.60 ± 1.70 mg/kg). The concentration of benzoic acid in the protein-85-product (Figure 4b) was similar to that of hemp seed flour (6.30 mg/kg) [33]. In this study [33], alkali and acid hydrolysis were used to extract the plant metabolites [33]. The amounts of benzoic acid in the protein-85-product (Figure 4b) were identified to be twice as low as those found in another study [78] on hemp seeds (19.4 mg/kg). The second most abundant plant metabolite (*p* < 0.01) in the protein-46-product was gentisic acid (46.2 ± 1.84 mg/kg). Gentisic acid in the protein-46-product (Figure 4b) was identified in similar amounts compared to a previous work on hemp seed flour (31.6 mg/kg) [33]. p-Coumaric acid was the second most abundant plant metabolite (*p* < 0.05) in hemp seed-hull flour (153 ± 5.37 mg/kg). The second most abundant plant metabolite in expellers was p-coumaric acid (113 mg/kg). p-Coumaric acid in expellers (Figure 4b) was found in similar amounts to those of a study on hemp cake (87.3 mg/kg), which used subcritical water extraction to obtain the plant metabolites [76]. The second most abundant plant metabolite in the cream solid residue (wet) was ferulic acid (31.4 ± 2.03 mg/kg). Ferulic acid in the cream solid residue (wet), as shown in Figure 4b, was similar to the amounts found on hemp seeds (52.0 mg/kg) in another study, which employed aqueous acetone (50%) to extract the plant metabolites [79]. Ferulic acid was the second most abundant plant metabolite (*p* < 0.01) in hemp seed hearts (18.8 ± 1.62 mg/kg), with a concentration (Figure 4b) similar to that of hemp seed flour (21.9 mg/kg) [33]. Syringaresinol was the second most abundant plant metabolite (*p* < 0.01) in protein fiber boost (54.6 ± 2.24 mg/kg). Syringaresinol was identified as the second most abundant plant metabolite in seeds (73.2 ± 4.26 mg/kg).

The concentration of the most abundant and second most abundant plant metabolites identified in the hemp seed-based samples (Figure 4b), in contrast to those reported in hemp screenings, hemp seeds, and hemp oil [74,75,77,78], may vary according to the type of extraction technique employed (alkaline hydrolysis, acid hydrolysis, and enzymatic hydrolysis), hemp seed variety, harvesting method, and geographical source of the raw material.

Based on the phytochemical distribution among the free and bound fractions, it can be seen that the plant metabolites are predominantly released following hydrolysis and found therefore bound to other components [80,81]. This potentially implies that the bioavailability of these plant metabolites in the upper gastrointestinal (GI) tract may be limited, and therefore, they may be delivered to the colon, where the can be released and metabolized into distinct types of molecules by the gut microbiota.

#### 3.3.2. Potential Health Benefits of Phytochemicals Identified in Hemp Seed-Based Samples and the Relevance of Guiding Possible Applications Within the Food Industry

Many of the plant metabolites analyzed in the hemp seed-based samples have been reported to have potential health benefits. For instance, the administration of gentisic acid (2.00 mg/mL) and syringin (20.0 μM) has been shown to effectively prevent obesity in mice and lipid accumulation in 3T3-L1 cells [82,83]. Treatments with vanillic acid (200 mg/kg) and p-hydroxybenzoic acid (40.0 mg/kg) have been reported to exhibit anti-inflammatory effects in mice [84,85,86]. Syringaresinol (100 μM) in Caco-2 cells lines has been shown to reduce tumor necrosis factor-α (TNF-α) and interleukin-6 (IL-6) [87]. The use of p-coumaric acid (100 μM) has been found to modulate glucose and lipid metabolism in L6 rat skeletal muscle cells [88]. Moreover, p-coumaric acid (1500 µM) has been shown to contain anti-proliferative effects on Caco-2 human cancer cells by decreasing their growth by 75% compared to controls after a 3-day treatment [89].

The food industry consistently strives to enhance the quality and health-related attributes of their products throughout processing and storage [90]. In terms of quality, lipid oxidation is widely recognized as the most crucial factor in food products, as it is responsible for undesirable characteristics, such as nutrient depletion, flavor and taste deterioration, and reduction in shelf life [91,92]. Consequently, these factors result in significant economic losses for food companies. As a solution, synthetic additives containing antioxidant activity have been used to control this process. Nevertheless, the use of synthetic additives has led to adverse effects, prompting a growing consumer preference for natural alternatives [93]. Several studies have highlighted the positive impact of plant extracts containing high levels of natural antioxidants on the regulation of oxidative processes in meat and meat products [90]. The hemp seed-based foods and their by-products from the present study are found to be rich in cinnamic acids and benzoic acids and several flavonoids, offering further applicability in the food industry as natural additives to potentially replace nitrites used for the preservation of food products. Therefore, the incorporation of plant derived-extracts abundant in various metabolites appears to be a feasible solution to address this concern.

## 4. Conclusions

In conclusion, this study shows that hemp seed-based foods and by-products are rich sources of protein and fiber and are particularly rich in micronutrients, including potassium, magnesium, and phosphorus, and bioactive phytochemicals, particularly p-coumaric acid, gentisic acid, syringaresinol, p-hydroxybenzaldehyde, benzoic acid, and ferulic acid. Almost all the hemp seed-based samples have the potential to deliver the recommended daily reference nutrient intake for several micronutrients, including magnesium, phosphorus, manganese, and iron. One hundred grams of all the hemp seed-based samples delivered the recommended daily intake for fatty acids, including linoleic acid and alpha-linolenic acid. Furthermore, the omega-6:omega-3 ratio found in all the hemp seed-based foods and in all the by-products was between 3:1 and 4:1 across all the samples analyzed and was not altered during the food processing of hemp seed. Therefore, the findings of this study support the consumption of hemp seed foods as part of the diet to diversify and help meet dietary recommendations. Furthermore, the findings support the revalorization of hemp by-products for food, which will help towards circular nutrition and deliver a truly green production of hemp food. The results of this study support the health-beneficial potential of hemp samples. However, it is essential to conduct a sensorial evaluation and assess the acceptability of consumers towards hemp seed-based samples, particularly those derived from by-products. Moreover, it would be important to evaluate the nutrient and bioactive compound metabolism and bioavailability in vivo to help properly understand their potential health benefits for the development of functional foods. Future human intervention studies should also assess the use of hemp-rich foods as part of nutritional therapies for the prevention of development of non-communicable diseases, such as type 2 diabetes and cardiovascular disease.

## Figures and Tables

**Table 1:** Macronutrient content of hemp seed-based foods and by-products: protein, total fat, and soluble and insoluble NSPs, expressed as mean % of the dry weight ± SD (*n* = 3).

Sample Type	Macronutrient Content (% Dry Weight)					
	Protein	Total Fat	Soluble NSPs	Insoluble NSPs	Ash	Moisture Content
**Protein fiber boost**	38.15 ± 0.13 ^d^	16.62 ± 0.19 ^f^	0.73 ± 0.19 ^a^	19.20 ± 0.66 ^d^	5.92 ± 0.03 ^c^	8.33 ± 0.08 ^cd^
**Protein-75-product**	74.50 ± 0.29 ^b^	10.91 ± 0.18 ^h^	0.33 ± 0.04 ^b^	4.27 ± 0.05 ^h^	11.74 ± 0.03 ^a^	6.29 ± 0.01 ^f^
**Protein-85-product**	93.01 ± 0.18 ^a^	3.24 ± 0.18 ^j^	0.43 ± 0.11 ^b^	3.91 ± 0.06 ^h^	7.76 ± 0.32 ^b^	4.72 ± 0.09 ^g^
**Protein-46-product**	55.62 ± 0.14 ^c^	13.44 ± 0.35 ^g^	0.62 ± 0.19 ^ab^	13.41 ± 0.46 ^f^	7.30 ± 0.02 ^b^	7.33 ± 0.08 ^e^
**Hemp seed-hull flour**	13.57 ± 0.10 ^i^	8.52 ± 0.13 ^i^	0.49 ± 0.03 ^ab^	39.80 ± 0.07 ^a^	2.33 ± 0.02 ^g^	8.42 ± 0.02 ^c^
**Hemp seed hearts**	35.87 ± 0.42 ^e^	45.51 ± 0.60 ^c^	0.45 ± 0.10 ^ab^	3.60 ± 0.08 ^h^	4.45 ± 0.02 ^e^	6.01 ± 0.04 ^f^
**Expellers**	25.13 ± 0.19 ^h^	10.10 ± 0.59 ^hi^	0.50 ± 0.02 ^ab^	31.60 ± 0.81 ^b^	3.58 ± 0.03 ^f^	7.87 ± 0.04 ^d^
**Seeds**	26.67 ± 0.19 ^g^	30.93 ± 1.60 ^e^	0.48 ± 0.02 ^ab^	17.50 ± 0.65 ^e^	3.41 ± 0.02 ^f^	8.47 ± 0.11 ^c^
**Hemp cake**	34.44 ± 0.19 ^f^	10.76 ± 0.18 ^h^	0.45 ± 0.07 ^ab^	24.20 ± 0.17 ^c^	4.38 ± 0.01 ^e^	9.70 ± 0.05 ^b^
**Hemp fudge**	37.66 ± 0.37 ^d^	45.10 ± 1.12 ^c^	0.40 ± 0.05 ^b^	4.81 ± 0.09 ^h^	5.04 ± 0.04 ^d^	5.12 ± 0.23 ^g^
**Cream solid residue (wet)**	35.60 ± 0.29 ^e^	41.34 ± 0.39 ^d^	0.40 ± 0.01 ^b^	8.13 ± 0.29 ^g^	4.34 ± 0.46 ^e^	2.85 ± 0.05 ^h^
**Cream solid residue (dried)**	35.91 ± 0.04 ^e^	42.22 ± 0.23 ^d^	0.40 ± 0.07 ^b^	8.63 ± 0.17 ^g^	4.36 ± 0.12 ^e^	2.88 ± 0.05 ^h^
**Hemp oil**	n/q	93.80 ± 0.89 ^a^	n/q	n/q	n/q	n/q
**Hemp cream**	n/q	63.10 ± 0.83 ^b^	n/q	n/q	0.20 ± 0.01 ^h^	19.40 ± 0.52 ^a^

Data within the same column with different letters are significantly different (*p* < 0.05). Where n/q = not quantified (i.e., samples were not analyzed for a particular macronutrient). NSP (non-starch polysaccharide). Water in cream solid residue (wet) was completely removed by freeze-drying. Water in cream solid residue (dried) was partially separated by centrifugation and completely removed by freeze-drying.

**Table 2:** Soluble and insoluble non starch polysaccharide (NSP) content and monosaccharide composition of the hemp seed-based foods and by-products expressed as mean % of the dry weight ± SD (*n* = 3).

Sample Type	NSP Type	Monosaccharide Content of NSP (% DM)								Total NSPs (% DM)
		Rhamnose	Fucose	Arabinose	Xylose	Mannose	Galactose	Glucose	Uronic acid	
**Protein fiber boost**	Soluble	0.03 ± 0.03 ^c^	0.04 ± 0.01 ^bc^	0.06 ± 0.03 ^bc^	0.07 ± 0.02 ^bc^	0.26 ± 0.02 ^a^	0.07 ± 0.02 ^bc^	0.05 ± 0.00 ^bc^	0.12 ± 0.04 ^b^	0.73
	Insoluble	0.38 ± 0.00 ^e^	0.10 ± 0.01 ^e^	1.38 ± 0.03 ^d^	7.07 ± 0.16 ^a^	0.46 ± 0.02 ^e^	0.71 ± 0.02 ^de^	5.94 ± 0.66 ^b^	3.15 ± 0.19 ^c^	19.20
**Protein-75-product**	Soluble	n/d	0.02 ± 0.00 ^b^	0.01 ± 0.00 ^b^	0.01 ± 0.01 ^b^	0.22 ± 0.01 ^a^	n/d	0.034 ± 0.00 ^b^	0.02 ± 0.04 ^b^	0.33
	Insoluble	0.12 ± 0.00 ^e^	0.04 ± 0.00 ^f^	0.88 ± 0.00 ^b^	0.53 ± 0.00 ^c^	0.26 ± 0.00 ^d^	0.23 ± 0.00 ^d^	1.20 ± 0.00 ^a^	0.88 ± 0.05 ^b^	4.27
**Protein-85-product**	Soluble	n/d	0.02 ± 0.01 ^b^	0.01 ± 0.00 ^b^	0.01 ± 0.01 ^b^	0.21 ± 0.01 ^a^	0.01 ± 0.01 ^b^	0.03 ± 0.01 ^b^	0.09 ± 0.08 ^b^	0.43
	Insoluble	0.12 ± 0.01 ^e^	0.04 ± 0.00 ^e^	0.56 ± 0.04 ^c^	0.45 ± 0.04 ^c^	0.26 ± 0.01 ^d^	0.24 ± 0.01 ^d^	1.28 ± 0.07 ^a^	0.91 ± 0.07 ^b^	3.91
**Protein-46-product**	Soluble	0.04 ± 0.02 ^b^	0.04 ± 0.01 ^b^	0.05 ± 0.02 ^b^	0.06 ± 0.03 ^b^	0.26 ± 0.03 ^a^	0.06 ± 0.02 ^b^	0.06 ± 0.04 ^b^	0.02 ± 0.00 ^b^	0.62
	Insoluble	0.33 ± 0.03 ^fg^	0.11 ± 0.02 ^g^	1.63 ± 0.03 ^d^	3.02 ± 0.04 ^b^	0.47 ± 0.02 ^ef^	0.63 ± 0.03 ^e^	4.74 ± 0.27 ^a^	2.42 ± 0.05 ^c^	13.41
**Hemp seed-hull flour**	Soluble	0.01 ± 0.01 ^c^	0.04 ± 0.01 ^b^	0.03 ± 0.00 ^b^	0.04 ± 0.00 ^b^	0.24 ± 0.01 ^a^	0.03 ± 0.00 ^b^	0.04 ± 0.01 ^b^	0.02 ± 0.00 ^bc^	0.49
	Insoluble	0.53 ± 0.00 ^e^	0.07 ± 0.00 ^f^	0.73 ± 0.00 ^d^	23.90 ± 0.09 ^a^	0.54 ± 0.01 ^e^	0.69 ± 0.01 ^d^	9.27 ± 0.01 ^b^	4.11 ± 0.10 ^c^	39.80
**Hemp seed hearts**	Soluble	0.01 ± 0.01 ^b^	0.03 ± 0.00 ^b^	0.03 ± 0.01 ^b^	0.06 ± 0.06 ^b^	0.22 ± 0.01 ^a^	0.03 ± 0.01 ^b^	0.04 ± 0.01 ^b^	0.01 ± 0.00 ^b^	0.45
	Insoluble	0.10 ± 0.00 ^e^	0.03 ± 0.00 ^f^	0.74 ± 0.02 ^b^	0.61 ± 0.03 ^c^	0.24 ± 0.01 ^d^	0.19 ± 0.01 ^d^	1.00 ± 0.02 ^a^	0.73 ± 0.01 ^b^	3.60
**Expellers**	Soluble	0.02 ± 0.02 ^b^	0.04 ± 0.00 ^b^	0.03 ± 0.00 ^b^	0.04 ± 0.01 ^b^	0.24 ± 0.01 ^a^	0.03 ± 0.00 ^b^	0.03 ± 0.00 ^b^	0.02 ± 0.00 ^b^	0.50
	Insoluble	0.46 ± 0.00 ^de^	0.07 ± 0.00 ^e^	0.91 ± 0.00 ^d^	18.60 ± 0.28 ^a^	0.48 ± 0.02 ^de^	0.64 ± 0.01 ^de^	6.85 ± 0.59 ^b^	3.52 ± 0.13 ^c^	31.60
**Seeds**	Soluble	n/d	0.03 ± 0.00 ^b^	0.03 ± 0.03 ^b^	0.02 ± 0.01 ^b^	0.25 ± 0.01 ^a^	0.04 ± 0.00 ^b^	0.04 ± 0.00 ^b^	0.04 ± 0.01 ^b^	0.48
	Insoluble	0.28 ± 0.00 ^e^	0.05 ± 0.00 ^e^	0.80 ± 0.01 ^d^	9.64 ± 0.30 ^a^	0.37 ± 0.01 ^e^	0.45 ± 0.02 ^de^	3.80 ± 0.28 ^b^	2.10 ± 0.04 ^c^	17.50
**Hemp cake**	Soluble	n/d	0.04 ± 0.02 ^b^	0.03 ± 0.01 ^b^	0.02 ± 0.01 ^b^	0.25 ± 0.01 ^a^	0.02 ± 0.02 ^b^	0.02 ± 0.00 ^b^	0.04 ± 0.01 ^b^	0.45
	Insoluble	0.38 ± 0.01 ^e^	0.07 ± 0.00 ^f^	1.05 ± 0.01 ^d^	13.30 ± 0.18 ^a^	0.45 ± 0.02 ^e^	0.59 ± 0.01 ^e^	5.41 ± 0.05 ^b^	2.90 ± 0.10 ^c^	24.20
**Hemp fudge**	Soluble	n/d	0.03 ± 0.00 ^b^	0.02 ± 0.00 ^b^	0.01 ± 0.01 ^b^	0.22 ± 0.04 ^a^	0.01 ± 0.01 ^b^	0.03 ± 0.00 ^b^	0.03 ± 0.01 ^b^	0.40
	Insoluble	0.12 ± 0.02 ^fg^	0.03 ± 0.00 ^g^	0.57 ± 0.01 ^d^	1.22 ± 0.06 ^b^	0.25 ± 0.03 ^e^	0.19 ± 0.00 ^ef^	1.56 ± 0.07 ^a^	0.76 ± 0.01 ^c^	4.81
**Cream solid residue (wet)**	Soluble	n/d	0.03 ± 0.00 ^bc^	0.02 ± 0.00 ^c^	0.01 ± 0.00 ^c^	0.21 ± 0.01 ^a^	0.01 ± 0.01 ^c^	0.03 ± 0.00 ^c^	0.05 ± 0.01 ^b^	0.40
	Insoluble	0.22 ± 0.00 ^cd^	0.06 ± 0.00 ^d^	1.38 ± 0.01 ^b^	2.06 ± 0.08 ^a^	0.32 ± 0.01 ^c^	0.40 ± 0.00 ^c^	1.81 ± 0.21 ^a^	1.83 ± 0.10 ^a^	8.13
**Cream solid residue (dried)**	Soluble	n/d	0.04 ± 0.02 ^b^	0.02 ± 0.00 ^b^	0.02 ± 0.00 ^b^	0.24 ± 0.04 ^a^	0.01 ± 0.01 ^b^	0.03 ± 0.00 ^b^	0.02 ± 0.01 ^b^	0.40
	Insoluble	5.01 ± 0.01 ^e^	0.24 ± 0.00 ^f^	0.07 ± 0.03 ^c^	1.46 ± 0.09 ^ab^	2.02 ± 0.00 ^de^	0.34 ± 0.01 ^d^	0.42 ± 0.06 ^a^	2.11 ± 0.03 ^b^	8.63

Where n/d = not detected (i.e., below the detection level); DM = dry matter. Data within the same row with different letters are significantly different (*p* < 0.05).

**Table 3:** Fatty acid composition of the hemp seed-based foods and by-products measured as methyl esters (FAME) expressed as average % from the individual fat content ± SD (*n* = 3).

Sample Type	Fatty Acid Methyl Esters % from Total Fat						Total Fat (%)	Ratio ω-6/ω-3 (Omega-6/Omega-3)
	Palmitic Acid	Stearic Acid	Oleic Acid	Linoleic Acid	Gamma-Linolenic Acid	Alpha-Linolenic Acid		
**Protein fiber boost**	8.98 ± 0.04 ^b^	3.04 ± 0.03 ^efg^	14.19 ± 0.04 ^fg^	59.74 ± 0.09 ^bcd^	3.06 ± 0.00 ^ab^	15.80 ± 0.02 ^b^	16.62 ± 0.19 ^f^	3.97
**Protein-75-product**	7.40 ± 0.09 ^d^	3.33 ± 0.07 ^cd^	15.73 ± 0.43 ^cd^	57.82 ± 1.31 ^def^	2.63 ± 0.15 ^d^	16.31 ± 0.46 ^b^	10.91 ± 0.18 ^h^	3.71
**Protein-85-product**	10.41 ± 1.60 ^a^	4.24 ± 0.17 ^a^	16.94 ± 0.85 ^ab^	53.80 ± 2.02 ^h^	2.23 ± 0.15 ^e^	12.07 ± 0.66 ^e^	3.24 ± 0.18 ^j^	4.64
**Protein-46-product**	9.07 ± 0.10 ^ab^	3.61 ± 0.03 ^b^	15.73 ± 0.14 ^cd^	59.32 ± 0.05 ^bcd^	2.63 ± 0.01 ^d^	13.38 ± 0.13 ^d^	13.44 ± 0.35 ^g^	4.63
**Hemp seed-hull flour**	9.03 ± 0.12 ^ab^	3.18 ± 0.07 ^def^	15.70 ± 0.10 ^cd^	60.10 ± 0.03 ^bc^	2.71 ± 0.01 ^cd^	14.30 ± 0.19 ^c^	8.52 ± 0.13 ^i^	4.39
**Hemp seed hearts**	7.08 ± 0.06 ^de^	3.04 ± 0.03 ^efg^	15.10 ± 0.03 ^def^	59.38 ± 0.08 ^bcd^	2.70 ± 0.01 ^cd^	15.85 ± 0.03 ^b^	45.51 ± 0.60 ^c^	3.92
**Expellers**	8.86 ± 0.41 ^bc^	3.35 ± 0.01 ^bcd^	15.79 ± 0.35 ^cd^	59.29 ± 0.62 ^bcd^	2.65 ± 0.05 ^d^	14.10 ± 0.15 ^c^	10.10 ± 0.59 ^hi^	4.14
**Seeds**	7.46 ± 0.02 ^cd^	3.08 ± 0.03 ^efg^	15.40 ± 0.03 ^cde^	61.10 ± 0.10 ^b^	2.74 ± 0.01 ^cd^	16.10 ± 0.05 ^b^	30.93 ± 1.60 ^e^	3.96
**Hemp cake**	9.09 ± 0.18 ^ab^	3.51 ± 0.09 ^bc^	16.10 ± 0.08 ^bc^	60.71 ± 0.36 ^b^	2.62 ± 0.03 ^d^	14.86 ± 0.23 ^c^	10.76 ± 0.18 ^h^	4.27
**Hemp fudge**	7.11 ± 0.07 ^de^	2.98 ± 0.08 ^fg^	14.57 ± 0.06 ^efg^	58.57 ± 0.30 ^cde^	2.71 ± 0.02 ^cd^	15.10 ± 0.10 ^b^	45.10 ± 1.12 ^c^	3.83
**Cream solid** **residue (wet)**	7.97 ± 0.09 ^bcd^	3.28 ± 0.02 ^cde^	14.14 ± 0.36 ^fg^	55.50 ± 0.37 ^gh^	2.91 ± 0.01 ^bc^	14.38 ± 0.04 ^c^	41.34 ± 0.39 ^d^	4.06
**Cream solid** **residue (dried)**	7.81 ± 0.18 ^bcd^	2.91 ± 0.17 ^gh^	13.87 ± 0.21 ^g^	56.47 ± 0.17 ^efg^	2.57 ± 0.06 ^d^	14.69 ± 0.09 ^c^	42.22 ± 0.23 ^d^	4.02
**Hemp oil**	5.93 ± 0.36 ^e^	2.67 ± 0.02 ^h^	12.91 ± 0.21 ^h^	55.10 ± 0.54 ^fg^	3.05 ± 0.20 ^ab^	16.25 ± 0.25 ^b^	93.80 ± 0.89 ^a^	3.63
**Hemp cream**	7.71 ± 0.07 ^bcd^	3.45 ± 0.04 ^bc^	17.35 ± 0.07 ^a^	69.53 ± 0.45 ^a^	3.26 ± 0.02 ª	18.85 ± 0.14 ^a^	63.10 ± 0.83 ^b^	3.86

Data within the same column with different letters are significantly different (*p* < 0.05). The following compounds were only detected in the range of 0.01–1% in any of the hemp samples: myristic acid, pentadecylic acid, palmitoleic acid, trans-10-heptadecenoic acid, cis-10-heptadecenoic acid, arachidic acid, eicosenoic acid, conjugated linoleic acid, eicosadienoic acid, behenic acid, cetoleic acid, trichosanoic acid, eikosapentaenoic acid, and nervonic acid.

**Table 4:** Hemp seed-based foods and by-products microelement content expressed as mg/100 g dry weight ± SD (*n* = 3).

Sample Type	Microelement (mg/100 g Dry Weight)								
	Na	K	Ca	Mg	P	Mn	Fe	Cu	Zn
**Protein fiber boost**	0.08 ± 0.10 ^c^	1166.81 ± 17.10 ^b^	373.84 ± 5.88 ^a^	628.39 ± 8.48 ^c^	1296.89 ± 14.41 ^d^	19.17 ± 0.19 ^b^	23.68 ± 0.39 ^b^	1.73 ± 0.01 ^a^	8.13 ± 0.08 ^ef^
**Protein-75-product**	17.29 ± 0.17 ^a^	980.95 ± 15.10 ^d^	280.34 ± 3.11 ^b^	1581.36 ± 5.74 ^a^	3096.66 ± 115 ^a^	24.29 ± 0.20 ^a^	36.20 ± 0.18 ^a^	0.99 ± 0.01 ^fgh^	27.62 ± 0.28 ^a^
**Protein-85-product**	7.17 ± 0.04 ^b^	135.04 ± 1.82 ^h^	166.64 ± 3.22 ^e^	401.69 ± 7.42 ^fg^	1922.26 ± 28.36 ^b^	13.23 ± 0.76 ^c^	34.62 ± 0.59 ^a^	0.81 ± 0.02 ^h^	24.72 ± 0.36 ^b^
**Protein-46-product**	n/d	1431.00 ± 49.08 ^a^	282.25 ± 11.10 ^b^	818.60 ± 28.06 ^b^	1740.12 ± 54.10 ^c^	19.15 ± 0.37 ^b^	22.30 ± 0.43 ^b^	1.75 ± 0.03 ^a^	12.72 ± 0.36 ^c^
**Hemp seed-** **hull flour**	n/d	456.27 ± 36.37 ^g^	201.63 ± 19.37 ^cd^	143.50 ± 18.40 ^h^	220.31 ± 19.85 ^h^	9.38 ± 0.90 ^de^	10.52 ± 1.51 ^f^	0.91 ± 0.07 ^gh^	1.71 ± 0.21 ^h^
**Hemp seed** **hearts**	0.56 ± 0.98 ^c^	870.54 ± 19.72 ^de^	108.42 ± 2.92 ^f^	509.06 ± 9.57 ^de^	1081.56 ± 37.51 ^e^	7.55 ± 0.15 ^f^	10.75 ± 0.17 ^ef^	1.12 ± 0.03 ^ef^	8.42 ± 0.20 ^e^
**Expellers**	n/d	757.80 ± 31.61 ^ef^	208.21 ± 11.33 ^c^	332.05 ± 19.89 ^g^	642.00 ± 28.82 ^g^	10.90 ± 0.51 ^d^	10.22 ± 0.62 ^f^	1.29 ± 0.05 ^de^	4.79 ± 0.34 ^g^
**Seeds**	n/d	676.69 ± 13.45 ^f^	168.04 ± 9.94 ^de^	348.39 ± 10.29 ^g^	694.94 ± 13.74 ^g^	9.38 ± 0.47 ^de^	9.22 ± 0.27 ^f^	1.07 ± 0.04 ^fg^	5.23 ± 0.24 ^g^
**Hemp cake**	0.09 ± 0.15 ^c^	914.22 ± 22.36 ^d^	232.66 ± 6.27 ^c^	450.24 ± 22.34 ^ef^	886.53 ± 12.34 ^f^	13.43 ± 0.88 ^c^	12.76 ± 0.56 ^de^	1.48 ± 0.06 ^bc^	6.91 ± 0.37 ^f^
**hemp fudge**	n/d	985.16 ± 106 ^cd^	268.84 ± 28.21 ^b^	607.67 ± 58.92 ^c^	1267.16 ± 135 ^d^	8.94 ± 0.89 ^ef^	15.59 ± 1.45 ^c^	1.41 ± 0.13 ^cd^	9.24 ± 0.93 ^e^
**Cream solid residue (wet)**	7.54 ± 0.16 ^b^	1190.85 ± 5.04 ^b^	141.52 ± 3.04 ^ef^	519.30 ± 4.31 ^de^	1213.96 ± 14.66 ^de^	8.84 ± 0.08 ^ef^	14.48 ± 0.14 ^cd^	1.58 ± 0.01 ^abc^	11.10 ± 0.16 ^d^
**Cream solid residue (dried)**	6.64 ± 0.47 ^b^	1116.09 ± 97.15 ^bc^	159.85 ± 13.29 ^e^	562.20 ± 44.65 ^cd^	1292.29 ± 113 ^d^	10.12 ± 0.89 ^de^	16.39 ± 1.34 ^c^	1.60 ± 0.11 ^ab^	12.13 ± 1.06 ^cd^
**Hemp oil**	0.22 ± 0.18 ^c^	0.79 ± 0.18 ^i^	1.63 ± 0.85 ^g^	0.07 ± 0.01 ^i^	n/d	0.01 ± 0.00 ^g^	0.07 ± 0.01 ^g^	0.01 ± 0.00 ^i^	0.02 ± 0.04 ^i^
**Hemp cream**	0.81 ± 0.16 ^c^	105.64 ± 8.14 ^hi^	2.40 ± 0.07 ^g^	8.22 ± 0.47 ^i^	45.10 ± 3.94 ^hi^	0.05 ± 0.00 ^g^	0.21 ± 0.01 ^g^	0.16 ± 0.01 ^i^	0.04 ± 0.02 ^i^

Data within the same column with different letters are significantly different (*p* < 0.05). The microelements reference nutrient intake (RNI) in mg/100 g: Sodium (Na), 1600; Potassium (K), 3500; Calcium (Ca), 700; Magnesium (Mg), 300; Phosphorus (P), 540; Manganese (Mn), 2.30; Iron (Fe), 8.70; Copper (Cu) 1.20; and Zinc (Zn), 9.50.

## Data Availability

The original contributions presented in this study are included in the article/Supplementary Materials. Further inquiries can be directed to the corresponding author.

## Supplementary Materials

The following supporting information can be downloaded at: https://www.mdpi.com/article/10.3390/foods14050875/s1, Table S1: Plant metabolite content of protein fiber boost, protein-75-product, protein-85-product, protein-46-product, hemp seed-hull flour, and hemp seed hearts in mg/kg dry weight ± SD (*n* = 3); Table S2: Plant metabolite content of expellers, seeds, hemp cake, hemp fudge, cream solid residue (wet), and cream solid residue (dried) in mg/kg dry weight ± SD (*n* = 3); Table S3: Percentage of recommended dietary allowances of fatty acid methyl esters met by the hemp seed-based foods and the by-products in a consumption of 100 g ± SD (*n* = 3); Table S4: Percentage of the reference nutrient intake for micronutrient content met by the hemp seed-based foods and the by-products in the consumption of 100 g ± SD (*n* = 3).

## Abbreviations

ADI	Average daily intake
FAMEs	Fatty acid methyl esters
GC	Gas chromatography
GLP-1	Glucagon-like peptide 1
HCl	Hydrochloric acid
ICP-MS	Inductively couple plasma mass spectrometry
IS1	Internal standard 1
IS2	Internal standard 2
LC-MS/MS	Liquid chromatography-mass spectrometry
NSPs	Non-starch polysaccharides
PCA	Principal component analysis
RNI	Reference nutrient intake
ω-6/ω-3	Omega-6/omega-3
