# Supplementary material for: Hemp Seed-Based Foods and Processing By-Products Are Sustainable Rich Sources of Nutrients and Plant Metabolites Supporting Dietary Biodiversity, Health, and Nutritional Needs
Source: Foods. 2025 Mar 4;14(5):875. doi: 10.3390/foods14050875 (PMC11899665; doi:10.3390/foods14050875)
Supplement: Supplementary file 1 [file foods-14-00875-s001.zip › foods-3487029-supplementary.pdf]

Supplementary information

Table S1. Plant metabolite content of protein fibre boost, protein-75-product, protein-85-product, protein-46-product, hemp seed-hull flour, and hemp seed hearts in mg/kg dry weight ± SD (n = 3). Benzoic acids, **Set A**; benzaldehydes, benzenes, acetophenones, **Set B**; cinnamic acids and phenolic dimers, **Set C**; phenylpropionic acids, phenylacetic acids, mandelic acids, phenyllactic acids and phenypyruvic acids, **Set D**; indoles and tryptophan, **Set E**; flavonoids, isoflavonoids and coumarins, **Set F** and lignans, **Set G**.

**Set A**	Protein fibre boost		Protein-75-product		Protein-85-product		Protein-46-product		Hemp seed-hull flour		Hemp seed hearts	
	Free	Bound	Free	Bound	Free	Bound	Free	Bound	Free	Bound	Free	Bound
Salicylic Acid	14.6 ± 1.73	3.03 ± 0.631	4.63 ± 0.848	0.231 ± 0.0483	6.87 ± 1.37	0.173 ± 0.0921	11.6 ± 2.25	2.14 ± 0.187	28.9 ± 0.899	16.7 ± 1.33	7.01 ± 0.0573	1.58 ± 0.281
p-Hydroxybenzoic Acid	4.57 ± 0.632	8.51 ± 1.59	1.76 ± 0.295	3.06 ± 0.634	2.91 ± 0.691	1.91 ± n/d	2.66 ± 0.501	13.9 ± 0.918	12.2 ± 0.553	19.1 ± 1.74	2.19 ± 0.232	9.93 ± 0.368
2,3-Dihydroxybenzoic Acid	n/d	0.0637 ± 0.111	0.0938 ± 0.00744	n/d	0.111 ± 0.0281	n/d	0.0918 ± 0.0121	0.284 ± 0.0327	n/d	0.221 ± 0.00449	n/d	0.205 ± 0.0233
Gentisic Acid	0.401 ± 0.0801	15.6 ± 6.89	1.19 ± 0.266	13.9 ± 4.53	5.45 ± 2.04	28.1 ± 5.38	0.473 ± 0.173	45.7 ± 1.73	0.281 ± 0.0694	11.1 ± 0.343	0.529 ± 0.0993	33.6 ± 2.12

2,6-Dihydroxybenzoic Acid	0.123 ± 0.0278	n/d	0.119 ± 0.0235	n/d	0.125 ± 0.0114	n/d	0.153 ± 0.0394	n/d	0.261 ± 0.0476	0.0553 ± 0.0478	0.0961 ± 0.0191	n/d
Protocatechuic Acid	5.24 ± 0.759	2.81 ± 0.586	1.45 ± 0.304	0.517 ± 0.144	1.41 ± 0.391	0.431 ± 0.0631	3.33 ± 0.751	2.93 ± 0.128	2.98 ± 0.131	4.51 ± 0.351	0.511 ± 0.00895	0.855 ± 0.0269
o-Anisic Acid	n/d	n/d	n/d	n/d	n/d	n/d	n/d	n/d	n/d	n/d	n/d	n/d
Vanillic Acid	3.83 ± 0.484	13.6 ± 1.92	0.824 ± 0.171	1.18 ± 0.227	1.29 ± 0.715	0.601 ± 0.0675	2.17 ± 0.314	11.1 ± 0.328	4.49 ± 0.331	29.9 ± 2.75	1.21 ± 0.232	4.83 ± 0.173
Syringic Acid	1.44 ± 0.274	6.76 ± 0.885	0.0221 ± 0.0381	0.147 ± 0.0174	0.565 ± 0.932	0.0483 ± 0.0161	0.624 ± 0.166	3.94 ± 0.168	1.22 ± 0.0888	15.7 ± 1.31	0.115 ± 0.0506	0.783 ± 0.0923
Benzoic Acid	0.846 ± 0.263	2.55 ± 0.242	1.63 ± 0.721	1.14 ± 0.208	6.31 ± 1.66	2.28 ± 0.195	0.741 ± 0.202	4.81 ± 1.01	1.18 ± 0.302	2.71 ± 0.859	0.604 ± 0.392	2.45 ± 0.863

**Set B**	Protein fibre boost		Protein-75-product		Protein-85-product		Protein-46-product		Hemp seed-hull flour		Hemp seed hearts	
	Free	Bound	Free	Bound	Free	Bound	Free	Bound	Free	Bound	Free	Bound

p-Hydroxybenzaldehyde	1.98 ± 0.249	14.4 ± 1.09	2.42 ± 0.322	6.79 ± 1.35	2.64 ± 0.631	5.53 ± 0.821	1.25 ± 0.242	12.7 ± 1.11	10.6 ± 0.499	27.7 ± 2.76	0.278 ± 0.0601	3.61 ± 0.648
Protocatachaldehyde	2.94 ± 0.449	2.95 ± 0.358	1.01 ± 0.212	0.535 ± 0.212	0.853 ± 0.239	0.837 ± 0.241	2.11 ± 0.422	3.81 ± 0.958	1.71 ± 0.0483	5.46 ± 0.569	0.182 ± 0.0182	2.01 ± 0.601
Vanillin	2.47 ± 0.363	23.2 ± 2.39	0.339 ± 0.0459	1.83 ± 0.252	0.654 ± 0.243	1.72 ± 0.113	1.35 ± 0.197	13.1 ± 0.731	6.12 ± 0.326	15.9 ± 1.75	0.191 ± 0.0233	3.58 ± 0.582
Syringin	2.31 ± 0.324	17.2 ± 1.63	0.0886 ± 0.0164	0.697 ± 0.177	0.488 ± 0.726	0.306 ± 0.0499	1.01 ± 0.173	9.65 ± 0.0423	3.48 ± 0.071	41.4 ± 2.81	0.113 ± 0.0445	2.11 ± 0.521
3,4-Dimethoxybenzaldehyde	n/d	n/d	n/d	n/d	n/d	n/d	n/d	n/d	n/d	n/d	n/d	n/d
Phenol	n/d	2.15 ± 3.73	n/d	n/d	n/d	n/d	n/d	n/d	0.441 ± 0.765	11.1 ± 0.578	n/d	n/d
4-Hydroxyacetophenone	0.0494 ± 0.00913	0.482 ± 0.0438	0.0318 ± 0.00581	0.114 ± 0.0187	0.0359 ± 0.00888	0.0817 ± 0.00398	0.0354 ± 0.00795	0.418 ± 0.00523	0.0949 ± 0.00783	0.488 ± 0.0271	0.0277 ± 0.00331	0.158 ± 0.00535

4-Hydroxy-3-Methoxyacetophenone	0.0803 ± 0.0116	0.831 ± 0.0742	n/d	0.146 ± 0.00168	0.0261 ± 0.0258	0.176 ± 0.0346	0.0377 ± 0.00824	0.752 ± 0.0534	0.0927 ± 0.0105	1.12 ± 0.0969	0.0122 ± 0.0104	0.367 ± 0.0117
4-Hydroxy-3,5-Dimethoxyacetophenone	0.115 ± 0.0111	0.723 ± 0.106	n/d	n/d	n/d	n/d	0.021 ± 0.0346	0.536 ± 0.0324	0.0917 ± 0.00861	1.17 ± 0.167	n/d	0.118 ± 0.0213

**Set C**	Protein fibre boost		Protein-75-product		Protein-85-product		Protein-46-product		Hemp seed-hull flour		Hemp seed hearts	
	Free	Bound	Free	Bound	Free	Bound	Free	Bound	Free	Bound	Free	Bound
Cinnamic Acid	0.304 ± 0.0514	0.0533 ± 0.00446	0.197 ± 0.0426	n/d	0.105 ± 0.0241	n/d	0.234 ± 0.0654	0.0247 ± 0.0216	0.333 ± 0.0143	0.194 ± 0.0363	0.387 ± 0.0189	0.0364 ± 0.00461
m-Coumaric Acid	n/d	n/d	n/d	n/d	n/d	n/d	n/d	n/d	n/d	n/d	n/d	n/d
p-Coumaric Acid	1.76 ± 0.254	106 ± 2.83	0.531 ± 0.0811	4.41 ± 0.651	1.17 ± 0.603	2.52 ± 0.223	1.04 ± 0.202	61.6 ± 2.89	2.492 ± 0.148	151 ± 5.43	2.15 ± 1.25	5.55 ± 1.81
Caffeic Acid	0.116 ± 0.0281	0.695 ± 0.122	0.0288 ± 0.0261	0.0247 ± 0.0224	0.0732 ± 0.0383	0.0257 ± 0.0226	0.0816 ± 0.0271	0.556 ± 0.0574	0.0393 ± 0.0341	0.935 ± 0.0666	0.141 ± 0.0231	0.0914 ± 0.0262

Ferulic Acid	1.37 ± 0.292	14.1 ± 2.72	2.24 ± 0.302	4.41 ± 1.04	3.21 ± 1.13	5.36 ± 0.315	1.76 ± 0.335	24.7 ± 0.882	0.831 ± 0.0303	7.88 ± 0.784	4.63 ± 0.103	14.1 ± 1.54
Sinapic Acid	0.0706 ± 0.0613	1.07 ± 0.182	0.0518 ± 0.0455	0.218 ± 0.0684	n/d	0.237 ± 0.0369	n/d	1.23 ± 0.104	n/d	0.664 ± 0.0113	0.106 ± 0.0517	0.848 ± 0.113
4-Methoxycinnamic Acid	n/d	0.0464 ± 0.0803	n/d	n/d	n/d	n/d	n/d	n/d	n/d	0.261 ± 0.0724	n/d	n/d
Ferulic Dimer (8-5 linked)	n/d	n/d	n/d	n/d	n/d	n/d	n/d	n/d	n/d	0.432 ± 0.0335	n/d	n/d
**Set D**	Protein fibre boost		Protein-75-product		Protein-85-product		Protein-46-product		Hemp seed-hull flour		Hemp seed hearts	
	Free	Bound	Free	Bound	Free	Bound	Free	Bound	Free	Bound	Free	Bound
3-Hydroxyphenylpropionic Acid	n/d	0.445 ± 0.0581	n/d	n/d	n/d	n/d	n/d	0.218 ± 0.0385	n/d	0.443 ± 0.0379	n/d	n/d
3,4-Dihydroxyphenylpropionic Acid	n/d	n/d	n/d	n/d	n/d	n/d	n/d	n/d	n/d	0.281 ± 0.488	n/d	n/d

4-Hydroxy-3-methoxyphenylpropionic Acid	0.0995 ± 0.0889	0.861 ± 0.209	0.668 ± 0.131	0.241 ± 0.0577	4.06 ± 0.589	0.203 ± 0.0553	n/d	1.18 ± 0.0742	0.0537 ± 0.0931	0.836 ± 0.146	n/d	1.22 ± 0.128
Phenylacetic Acid	0.466 ± 0.0543	0.324 ± 0.0248	0.644 ± 0.111	0.125 ± 0.0445	0.661 ± 0.197	0.194 ± 0.0338	0.457 ± 0.0888	0.401 ± 0.0537	0.644 ± 0.0461	0.381 ± 0.0232	0.217 ± 0.0288	0.0895 ± 0.0211
4-Hydroxyphenylacetic Acid	0.221 ± 0.0558	1.37 ± 0.212	0.847 ± 0.124	0.454 ± 0.121	1.02 ± 0.322	n/d	0.269 ± 0.0639	2.57 ± 0.0865	0.284 ± 0.0144	2.28 ± 0.101	0.283 ± 0.0624	2.68 ± 0.302
Mandelic Acid	n/d	n/d	n/d	n/d	n/d	n/d	n/d	n/d	n/d	n/d	n/d	n/d
3-Hydroxymandelic Acid	0.159 ± 0.00660	0.137 ± 0.0212	n/d	n/d	0.0251 ± 0.0434	n/d	0.101 ± 0.0885	0.207 ± 0.0758	0.0889 ± 0.0142	0.155 ± 0.0307	n/d	n/d
4-Hydroxymandelic Acid	0.267 ± 0.0465	0.489 ± 0.227	0.0251 ± 0.044	n/d	n/d	n/d	0.582 ± 0.126	0.103 ± 0.0895	0.304 ± 0.0147	2.57 ± 0.227	0.613 ± 0.0182	n/d
3,4-Dihydroxymandelic Acid	0.653 ± 0.104	0.486 ± 0.0710	0.0823 ± 0.0785	n/d	0.0304 ± 0.0526	n/d	0.872 ± 0.0431	0.686 ± 0.0709	0.563 ± 0.0539	0.514 ± 0.0928	0.775 ± 0.0515	0.576 ± 0.0504

4-Hydroxy-3-Methoxymandelic Acid	n/d	n/d	n/d	n/d	n/d	n/d	n/d	n/d	n/d	5.45 ± 0.306	n/d	n/d
4-Hydroxyphenyllactic Acid	n/d	1.11 ± 0.540	0.931 ± 0.161	0.576 ± 0.181	3.71 ± 0.655	1.21 ± 0.0603	n/d	1.77 ± 0.0902	n/d	0.741 ± 0.0504	n/d	1.42 ± 0.139
Phenyllactic Acid	0.0511 ± 0.0123	n/d	2.27 ± 0.363	n/d	6.08 ± 1.31	0.0249 ± 0.0216	0.0446 ± 0.0136	0.0371 ± 0.00229	0.0546 ± 0.00492	0.0111 ± 0.0192	0.00834 ± 0.0144	0.0203 ± 0.0183
Phenylpyruvic Acid	0.152 ± 0.133	0.332 ± 0.0679	0.477 ± 0.169	0.169 ± 0.146	n/d	0.178 ± 0.154	0.292 ± 0.0685	0.271 ± 0.0167	n/d	0.0664 ± 0.115	n/d	n/d
4-Hydroxyphenylpyruvic Acid	1.15 ± 0.303	6.85 ± 1.53	0.979 ± 0.348	2.64 ± 0.0934	1.56 ± 0.563	2.59 ± 0.716	0.424 ± 0.371	1.57 ± 0.142	1.18 ± 0.162	6.96 ± 1.47	n/d	1.28 ± 0.128
**Set E**	Protein fibre boost		Protein-75-product		Protein-85-product		Protein-46-product		Hemp seed-hull flour		Hemp seed hearts	
	Free	Bound	Free	Bound	Free	Bound	Free	Bound	Free	Bound	Free	Bound
Indole	0.672 ± 0.0981	0.728 ± 0.0453	0.339 ± 0.0593	0.224 ± 0.0572	0.521 ± 0.119	0.242 ± 0.0155	0.306 ± 0.0824	0.705 ± 0.0327	1.42 ± 0.0995	1.24 ± 0.0825	n/d	0.261 ± 0.0312

Indole-3-Acetic Acid	n/d	n/d	0.0794 ± 0.0173	n/d	0.052 ± 0.0526	n/d	0.0814 ± 0.00946	0.0409 ± 0.0708	n/d	n/d	0.0253 ± 0.0439	n/d
Indole-3-Carboxylic Acid	0.865 ± 0.104	0.599 ± 0.0528	0.252 ± 0.0556	0.112 ± 0.0191	0.137 ± 0.0329	0.122 ± 0.0125	0.406 ± 0.0958	0.434 ± 0.0493	1.31 ± 0.0345	1.59 ± 0.121	0.175 ± 0.0168	0.241 ± 0.0519
Indole-3-Acrylic Acid	n/d	n/d	n/d	n/d	n/d	n/d	n/d	n/d	n/d	n/d	n/d	n/d
Indole-3-Pyruvic Acid	45.4 ± 31.2	140 ± 38.3	55.9 ± 4.08	100 ± 24.9	34.2 ± 4.31	134 ± 2.68	12.6 ± 4.97	116 ± 24.9	17.1 ± 3.46	71.9 ± 10.7	9.93 ± 3.62	61.6 ± 14.1
Indoe-3-Lactic Acid	0.0398 ± 0.691	0.0241 ± 0.0418	0.221 ± 0.0382	n/d	0.148 ± 0.142	n/d	n/d	n/d	n/d	n/d	0.0194 ± 0.0337	n/d
I3-Carboxaldehyde	0.491 ± 0.0726	0.601 ± 0.0835	0.297 ± 0.0414	0.234 ± 0.0473	0.512 ± 0.127	0.244 ± 0.0132	0.211 ± 0.0542	0.603 ± 0.0268	1.06 ± 0.0356	0.883 ± 0.0342	0.0489 ± 0.00252	0.233 ± 0.0197
Niacin	1.17 ± 0.118	7.57 ± 3.39	1.41 ± 0.198	6.71 ± 3.11	0.779 ± 0.254	3.46 ± 0.951	2.12 ± 0.633	12.6 ± 1.87	0.294 ± 0.0212	3.61 ± 0.327	1.56 ± 0.0341	9.78 ± 1.91

Kynurenic Acid	0.135 ± 0.0157	0.0740 ± 0.0650	0.0783 ± 0.0165	n/d	0.0441 ± 0.0383	0.0153 ± 0.0265	0.181 ± 0.0397	0.116 ± 0.00181	0.118 ± 0.00882	n/d	0.137 ± 0.0132	0.0643 ± 0.00894
**Set F**	Protein fibre boost		Protein-75-product		Protein-85-product		Protein-46-product		Hemp seed-hull flour		Hemp seed hearts	
	Free	Bound	Free	Bound	Free	Bound	Free	Bound	Free	Bound	Free	Bound
Ethylferulate	n/d	n/d	n/d	n/d	n/d	n/d	n/d	n/d	n/d	n/d	n/d	n/d
Chlorogenic Acid	2.99 ± 0.223	n/d	n/d	n/d	n/d	n/d	1.73 ± 0.354	n/d	1.09 ± 0.135	n/d	0.0794 ± 0.0112	n/d
Tyrosol	1.36 ± 0.204	2.81 ± 0.632	1.08 ± 0.202	1.43 ± 0.406	0.646 ± 0.271	0.429 ± 0.375	0.783 ± 0.0565	5.17 ± 0.515	1.59 ± 0.0581	6.96 ± 0.429	0.77 ± 0.0364	7.08 ± 1.07
Quinadilic Acid	0.0491 ± 0.0106	0.0774 ± 0.00400	0.0381 ± 0.00526	0.0623 ± 0.00173	0.0366 ± 0.00462	0.0648 ± 0.00192	0.0478 ± 0.0112	0.0718 ± 0.00241	0.0499 ± 0.00147	0.0866 ± 0.00272	0.771 ± 0.00297	0.0863 ± 0.00732
Anthranilic Acid	0.0583 ± 0.00725	0.626 ± 0.0681	n/d	0.0895 ± 0.00649	0.0181 ± 0.0328	0.133 ± 0.0744	0.0591 ± 0.00582	0.513 ± 0.172	0.0347 ± 0.0304	0.634 ± 0.0292	0.0555 ± 0.00672	0.558 ± 0.124

4-Ethylphenol	n/d	n/d	n/d	n/d	n/d	n/d	n/d	n/d	n/d	n/d	n/d	n/d
Coniferyl Alcohol	0.420 ± 0.0584	n/d	0.673 ± 0.287	0.0895 ± 0.0776	0.709 ± 0.135	0.131 ± 0.134	0.163 ± 0.282	n/d	0.529 ± 0.476	n/d	0.173 ± 0.152	n/d
Catechin	0.819 ± 0.209	n/d	n/d	n/d	n/d	n/d	n/d	n/d	n/d	n/d	n/d	n/d
Epicatechin	n/d	n/d	n/d	n/d	n/d	n/d	n/d	n/d	n/d	n/d	n/d	n/d
Isoliquiritigenin	n/d	n/d	n/d	n/d	n/d	n/d	n/d	n/d	n/d	n/d	n/d	n/d
Naringenin	n/d	n/d	n/d	n/d	n/d	n/d	n/d	n/d	n/d	n/d	n/d	n/d
Naringin	0.218 ± 0.0135	n/d	0.0506 ± 0.0441	n/d	n/d	n/d	0.153 ± 0.0245	n/d	0.151 ± 0.00394	n/d	0.187 ± 0.00634	n/d

Kaempferol	n/d	n/d	n/d	n/d	n/d	n/d	n/d	n/d	n/d	n/d	n/d	n/d
Morin	n/d	n/d	n/d	n/d	n/d	n/d	n/d	n/d	n/d	n/d	n/d	n/d
Quercetin	0.101 ± 0.0909	n/d	0.133 ± 0.0451	n/d	0.0355 ± 0.0616	n/d	n/d	n/d	0.0595 ± 0.0517	n/d	n/d	n/d
Myricetin	0.0985 ± 0.170	n/d	n/d	n/d	n/d	n/d	n/d	n/d	n/d	n/d	n/d	n/d
Quercetin-3-Glucoside	0.849 ± 0.123	0.00158 ± 0.00273	0.0117 ± 0.00273	n/d	0.0177 ± 0.00643	n/d	0.277 ± 0.0607	n/d	0.688 ± 0.0131	n/d	0.00191 ± 0.00331	n/d
Taxifolin	0.160 ± 0.0290	n/d	n/d	n/d	n/d	n/d	0.0495 ± 0.0461	n/d	0.0763 ± 0.0157	n/d	n/d	n/d
Genistein	0.344 ± 0.0256	0.0363 ± 0.0629	0.138 ± 0.00458	0.0332 ± 0.0576	0.181 ± 0.0217	n/d	0.157 ± 0.0176	n/d	0.171 ± 0.00423	n/d	n/d	n/d

Scopoletin	n/d	n/d	n/d	n/d	n/d	n/d	n/d	n/d	n/d	n/d	n/d	n/d
Quercitrin	0.232 ± 0.205	0.0902 ± 0.117	n/d	n/d	0.00823 ± 0.0142	n/d	0.0539 ± 0.0181	n/d	n/d	n/d	n/d	n/d
Luteolin	1.52 ± 0.700	0.102 ± 0.177	0.453 ± 0.0631	n/d	0.428 ± 0.0863	n/d	0.387 ± 0.0635	n/d	0.475 ± 0.0817	n/d	n/d	0.343 ± 0.594
Formononetin	0.00728 ± 0.0126	n/d	n/d	n/d	n/d	n/d	n/d	n/d	0.00535 ± 0.00927	n/d	n/d	n/d
Apigenin	0.301 ± 0.0344	0.0986 ± 0.00232	0.121 ± 0.00586	0.0878 ± 0.00195	0.156 ± 0.0191	0.0878 ± 0.00156	0.136 ± 0.0124	0.144 ± 0.0483	0.158 ± 0.00931	0.0311 ± 0.0538	0.0847 ± 0.00121	0.0296 ± 0.0514
Luteolinidin	n/d	n/d	n/d	n/d	n/d	n/d	n/d	n/d	n/d	0.0188 ± 0.0327	n/d	n/d
Coumesterol	0.0426 ± 0.00436	0.0886 ± 0.00474	0.0415 ± 0.00143	0.0812 ± 0.00171	0.0407 ± 0.00286	0.0763 ± 0.00123	0.0246 ± 0.0215	0.0372 ± 0.00247	n/d	0.0128 ± 0.0221	0.0129 ± 0.0223	0.0117 ± 0.0203

8-Methylpsoralen	0.0110 ± 0.00162	0.00340 ± 0.00591	0.00313 ± 0.00543	0.00646 ± 0.0111	0.00343 ± 0.00594	n/d	n/d	n/d	n/d	n/d	n/d	n/d
Bergapten	0.00937 ± 0.00842	0.0129 ± 0.0129	0.00384 ± 0.00666	0.0151 ± 0.00696	0.00376 ± 0.00652	0.00371 ± 0.00642	0.00354 ± 0.00613	n/d	n/d	n/d	n/d	n/d
Tangeretin	0.0361 ± 0.00428	0.0764 ± 0.00184	0.0284 ± 0.00217	0.0571 ± 0.00174	0.0258 ± 0.00376	0.0468 ± 0.00391	0.0202 ± 0.00154	0.0302 ± 0.00424	0.0189 ± 0.00197	0.0366 ± 0.00262	0.0159 ± 0.000637	0.0221 ± 0.00104
Imperatorin	0.0223 ± 0.000847	0.0452 ± 0.00203	0.0227 ± 0.00348	0.0427 ± 0.000651	0.0194 ± n/d	0.0384 ± 0.000875	0.0186 ± 0.00104	0.0372 ± 0.00195	0.0193 ± 0.00122	0.0376 ± 0.00218	0.0121 ± 0.0106	n/d
Rutin	0.161 ± 0.0157	n/d	n/d	n/d	n/d	n/d	0.115 ± 0.0512	n/d	0.0916 ± 0.00562	n/d	n/d	n/d
Isorhamnetin	n/d	n/d	n/d	n/d	n/d	n/d	n/d	n/d	n/d	n/d	n/d	n/d
Vitexin	0.571 ± 0.0436	1.81 ± 0.384	0.123 ± 0.0136	0.328 ± 0.0621	0.133 ± 0.0643	0.334 ± 0.0407	0.271 ± 0.0414	1.81 ± 0.122	0.315 ± 0.0225	1.32 ± 0.0886	0.0707 ± 0.00243	0.507 ± 0.0435

Hyperoside	0.853 ± 0.104	0.00900 ± 0.00782	0.0164 ± 0.00255	n/d	0.0231 ± 0.00413	n/d	0.276 ± 0.0696	n/d	0.672 ± 0.00221	0.00861 ± 0.00823	0.00658 ± 0.00555	n/d
Glycitein	0.0361 ± 0.00229	0.0334 ± 0.00139	0.0323 ± 0.000588	0.0681 ± 0.00117	0.0352 ± 0.00112	0.0631 ± 0.000822	0.0315 ± 0.00128	0.0306 ± 0.00125	0.0203 ± 0.0176	n/d	0.0296 ± 0.000411	0.0608 ± 0.00236

**Set G**	Protein fibre boost		Protein-75-product		Protein-85-product		Protein-46-product		Hemp seed-hull flour		Hemp seed hearts	
	Free	Bound	Free	Bound	Free	Bound	Free	Bound	Free	Bound	Free	Bound
Secoisolariciresinol	n/d	0.455 ± 0.0982	n/d	n/d	n/d	n/d	n/d	0.221 ± 0.00985	n/d	1.71 ± 0.158	n/d	n/d
Syringaresinol	n/d	54.6 ± 2.24	n/d	n/d	1.15 ± 2.01	n/d	n/d	13.8 ± 3.85	1.31 ± 1.14	171 ± 16.1	n/d	n/d
Pinoresinol	0.154 ± 0.0176	0.462 ± 0.0255	0.0614 ± 0.0111	n/d	0.228 ± 0.0446	n/d	0.0582 ± 0.0191	0.0761 ± 0.0113	0.175 ± 0.00412	1.6 ± 0.137	0.00484 ± 0.00838	n/d

Where n/d = not detected (i.e., below the detection level).

Table S2. Plant metabolite content of expellers, seeds, hemp cake, hemp fudge, cream solid residue (wet), and cream solid residue (dried) in mg/kg dry weight ± SD (n = 3). Benzoic acids, **Set A**; benzaldehydes, benzenes, acetophenones, **Set B**; cinnamic acids and phenolic dimers, **Set C**; phenylpropionic acids, phenylacetic acids, mandelic acids, phenyllactic acids and phenypyruvic acids, **Set D**; indoles and tryptophan, **Set E**; flavonoids, isoflavonoids and coumarins, **Set F** and lignans, **Set G**.

**Set A**	Expellers		Seeds		Hemp cake		Hemp fudge		Cream solid residue (wet)		Cream solid residue (dried)	
	Free	Bound	Free	Bound	Free	Bound	Free	Bound	Free	Bound	Free	Bound
Salicylic Acid	23.3 ± 1.27	17.3 ± 0.847	17.1 ± 0.749	10.3 ± 1.02	23.5 ± 1.47	15.8 ± 1.91	9.41 ± 0.934	2.67 ± 0.604	6.19 ± 0.618	0.886 ± 0.0523	5.19 ± 0.300	0.627 ± 0.0239
p-Hydroxybenzoic Acid	8.59 ± 0.613	19.6 ± 0.533	5.86 ± 0.456	14.9 ± 0.831	7.72 ± 0.441	21.6 ± 1.28	2.14 ± 0.305	12.1 ± 2.03	2.74 ± 0.472	15.7 ± 1.57	1.92 ± 0.0931	10.5 ± 0.384
2,3-Dihydroxybenzoic Acid	n/d	0.312 ± 0.0313	n/d	0.277 ± 0.0212	n/d	0.378 ± 0.0654	n/d	0.294 ± 0.0644	0.145 ± 0.251	0.212 ± 0.0241	n/d	0.124 ± 0.0175
Gentisic Acid	0.351 ± 0.121	25.1 ± 3.23	0.209 ± 0.0491	28.8 ± 2.26	0.624 ± 0.0707	41.6 ± 6.78	0.391 ± 0.0446	37.5 ± 6.22	0.731 ± 0.411	35.8 ± 2.39	0.383 ± 0.0296	25.5 ± 2.19
2,6-Dihydroxybenzoic Acid	0.327 ± 0.0461	0.101 ± 0.00762	0.174 ± 0.0117	n/d	0.314 ± 0.0394	0.0967 ± 0.0905	0.146 ± 0.0191	n/d	0.0744 ± 0.0138	n/d	0.0415 ± 0.00499	n/d

Protocatechuic Acid	2.01 ± 0.0909	5.51 ± 0.326	1.24 ± 0.0643	4.44 ± 0.375	2.51 ± 0.128	5.86 ± 0.496	0.952 ± 0.133	2.35 ± 0.346	0.664 ± 0.584	1.51 ± 0.186	0.892 ± 0.0845	1.17 ± 0.0682
o-Anisic Acid	n/d	n/d	n/d	n/d	n/d	0.236 ± 0.0186	n/d	0.227 ± 0.0152	n/d	n/d	n/d	n/d
Vanillic Acid	3.91 ± 0.0935	29.1 ± 2.14	3.07 ± 0.248	19.1 ± 1.64	4.28 ± 0.283	26.1 ± 1.36	1.62 ± 0.253	7.74 ± 1.18	1.22 ± 0.222	7.58 ± 0.134	0.842 ± 0.0891	5.15 ± 0.184
Syringic Acid	0.836 ± 0.0485	15.1 ± 0.467	0.654 ± 0.0394	9.11 ± 0.757	1.02 ± 0.0364	12.6 ± 1.81	0.337 ± 0.0831	2.25 ± 0.352	0.234 ± 0.0577	1.91 ± 0.0898	0.0933 ± 0.0821	1.36 ± 0.132
Benzoic Acid	1.33 ± 0.431	4.55 ± 0.585	0.911 ± 0.562	2.43 ± 1.36	1.88 ± 0.338	4.58 ± 0.756	0.816 ± 0.279	3.43 ± 0.384	3.51 ± 0.411	8.42 ± 1.33	2.99 ± 0.0897	8.16 ± 0.681
**Set B**	Expellers		Seeds		Hemp cake		Hemp fudge		Cream solid residue (wet)		Cream solid residue (dried)	
	Free	Bound	Free	Bound	Free	Bound	Free	Bound	Free	Bound	Free	Bound
p-Hydroxybenzaldehyde	5.68 ± 0.372	22.2 ± 2.09	1.71 ± 0.0544	12.6 ± 1.59	2.95 ± 0.209	19.6 ± 1.93	1.22 ± 0.134	6.89 ± 0.743	0.712 ± 0.0795	14.3 ± 1.54	0.664 ± 0.0671	13.5 ± 0.842

Protocatachaldehyde	1.11 ± 0.0648	5.58 ± 0.387	0.575 ± 0.0476	6.84 ± 0.317	1.06 ± 0.108	6.41 ± 0.682	0.727 ± 0.121	5.31 ± 0.793	0.617 ± 0.0951	1.92 ± 0.244	0.555 ± 0.0275	1.71 ± 0.121
Vanillin	4.37 ± 0.0526	29.1 ± 24.3	1.94 ± 0.0583	29.5 ± 2.91	3.52 ± 0.323	49.1 ± 4.16	0.687 ± 0.104	8.41 ± 1.08	0.437 ± 0.0245	9.11 ± 0.748	0.333 ± 0.0249	8.18 ± 0.102
Syringin	2.49 ± 0.0362	32.7 ± 2.95	1.21 ± 0.0306	18.7 ± 1.71	2.33 ± 0.127	29.2 ± 1.91	0.525 ± 0.0928	6.11 ± 0.665	0.0808 ± 0.00516	4.93 ± 0.495	0.0697 ± 0.00327	3.93 ± 0.158
3,4-Dimethoxybenzaldehyde	n/d	n/d	n/d	n/d	n/d	n/d	n/d	n/d	n/d	n/d	n/d	0.0339 ± 0.0307
Phenol	n/d	14.5 ± 2.67	n/d	8.93 ± 0.438	n/d	10.8 ± 1.89	n/d	n/d	n/d	n/d	n/d	n/d
4-Hydroxyacetophenone	0.0688 ± 0.00242	0.476 ± 0.0162	0.0292 ± 0.00487	0.394 ± 0.00274	0.0425 ± 0.00192	0.524 ± 0.0114	0.0401 ± 0.00947	0.333 ± 0.0258	0.0119 ± 0.00207	0.219 ± 0.0257	0.00535 ± 0.00141	0.148 ± 0.00645
4-Hydroxy-3-Methoxyacetophenone	0.0859 ± 0.00463	1.14 ± 0.0355	0.0411 ± 0.00291	0.856 ± 0.0255	0.0746 ± 0.00452	1.16 ± 0.137	0.0274 ± 0.00697	0.541 ± 0.0398	0.00996 ± 0.00899	0.551 ± 0.0410	0.000836 ± 0.00144	0.432 ± 0.0248

4-Hydroxy-3,5-Dimethoxyacetophenone	0.0884 ± 0.00661	1.03 ± 0.0805	0.0442 ± 0.0386	0.678 ± 0.0434	0.0807 ± 0.0708	1.01 ± 0.0611	n/d	0.282 ± 0.0841	n/d	0.288 ± 0.0421	n/d	0.216 ± 0.0248
**Set C**	Expellers		Seeds		Hemp cake		Hemp fudge		Cream solid residue (wet)		Cream solid residue (dried)	
	Free	Bound	Free	Bound	Free	Bound	Free	Bound	Free	Bound	Free	Bound
Cinnamic Acid	0.556 ± 0.0156	0.231 ± 0.0285	0.322 ± 0.0284	0.141 ± 0.0249	0.601 ± 0.0684	0.241 ± 0.0735	0.527 ± 0.0622	0.0507 ± 0.0181	0.388 ± 0.0457	n/d	0.333 ± 0.0123	n/d
m-Coumaric Acid	n/d	n/d	n/d	n/d	n/d	0.0361 ± 0.0429	n/d	0.0232 ± 0.0312	n/d	n/d	n/d	n/d
p-Coumaric Acid	1.91 ± 0.442	112 ± 2.51	1.35 ± 0.112	80.3 ± 11.2	1.86 ± 0.143	114 ± 7.61	0.758 ± 0.101	35.7 ± 3.82	0.705 ± 0.0925	28.7 ± 8.64	0.516 ± 0.0265	18.5 ± 1.32
Caffeic Acid	0.0747 ± 0.0139	0.723 ± 0.0312	0.0974 ± 0.0133	0.533 ± 0.0667	0.166 ± 0.0255	1.01 ± 0.0483	0.0741 ± 0.00473	0.418 ± 0.0883	0.0876 ± 0.0219	0.331 ± 0.0759	0.0403 ± 0.0349	0.205 ± 0.0527
Ferulic Acid	2.46 ± 0.0565	12.5 ± 0.885	2.77 ± 0.422	12.7 ± 0.491	4.62 ± 0.152	21.8 ± 2.13	3.14 ± 0.312	18.9 ± 3.07	3.43 ± 0.467	28.1 ± 1.56	2.42 ± 0.183	17.4 ± 1.80

Sinapic Acid	0.0496 ± 0.0431	1.14 ± 0.0188	n/d	1.11 ± 0.148	0.0923 ± 0.0177	1.82 ± 0.0674	0.201 ± 0.0195	1.68 ± 0.199	0.244 ± 0.0597	1.58 ± 0.102	0.152 ± 0.0171	0.949 ± 0.127
4-Methoxycinnamic Acid	n/d	0.127 ± 0.0164	n/d	0.0337 ± 0.0584	n/d	0.109 ± 0.106	n/d	0.0437 ± 0.0757	n/d	n/d	n/d	n/d
Ferulic Dimer (8-5 linked)	n/d	0.353 ± 0.0526	n/d	0.233 ± 0.0202	n/d	0.393 ± 0.0281	n/d	n/d	n/d	n/d	n/d	n/d

**Set D**	Expellers		Seeds		Hemp cake		Hemp fudge		Cream solid residue (wet)		Cream solid residue (dried)	
	Free	Bound	Free	Bound	Free	Bound	Free	Bound	Free	Bound	Free	Bound
3-Hydroxyphenylpropionic Acid	n/d	0.245 ± 0.0323	n/d	0.214 ± 0.0591	n/d	0.341 ± 0.0634	n/d	0.0433 ± 0.0751	n/d	n/d	n/d	n/d
3,4-Dihydroxyphenylpropionic Acid	n/d	n/d	n/d	n/d	n/d	n/d	n/d	0.171 ± 0.297	n/d	0.197 ± 0.173	n/d	0.162 ± 0.280
4-Hydroxy-3-methoxyphenylpropionic Acid	0.0965 ± 0.0839	1.24 ± 0.169	0.0796 ± 0.0697	1.28 ± 0.0805	0.184 ± 0.0322	1.76 ± 0.432	0.0338 ± 0.0587	1.27 ± 0.311	0.0241 ± 0.0419	1.101 ± 0.140	n/d	0.705 ± 0.172

Phenylacetic Acid	0.573 ± 0.0303	0.374 ± 0.0428	0.478 ± 0.0451	0.275 ± 0.0662	0.706 ± 0.0393	0.482 ± 0.0564	0.358 ± 0.0638	0.243 ± 0.0367	0.246 ± 0.0183	0.243 ± 0.0248	0.192 ± 0.0192	0.203 ± 0.0138
4-Hydroxyphenylacetic Acid	0.325 ± 0.0272	2.85 ± 0.236	0.0829 ± 0.143	2.58 ± 0.114	0.404 ± 0.0351	3.62 ± 0.332	n/d	2.68 ± 0.597	0.213 ± 0.0447	3.02 ± 0.211	n/d	1.94 ± 0.252
Mandelic Acid	n/d	n/d	n/d	n/d	n/d	n/d	n/d	n/d	n/d	3.02 ± 0.182	n/d	2.22 ± 0.0422
3-Hydroxymandelic Acid	0.0185 ± 0.0321	0.151 ± 0.00431	n/d	0.207 ± 0.0415	n/d	0.392 ± 0.0947	n/d	0.307 ± 0.0991	n/d	n/d	n/d	n/d
4-Hydroxymandelic Acid	0.491 ± 0.0126	1.45 ± 0.172	0.873 ± 0.0862	0.819 ± 0.112	1.01 ± 0.00983	1.29 ± 0.0594	0.212 ± 0.0422	n/d	0.222 ± 0.200	n/d	0.225 ± 0.0406	n/d
3,4-Dihydroxymandelic Acid	0.831 ± 0.0589	0.654 ± 0.0802	0.749 ± 0.0962	0.513 ± 0.127	1.01 ± 0.0295	0.765 ± 0.0312	0.768 ± 0.0332	0.683 ± 0.0774	0.600 ± 0.134	0.372 ± 0.0742	0.360 ± 0.0849	0.224 ± 0.00566
4-Hydroxy-3-Methoxymandelic Acid	n/d	4.53 ± 0.587	n/d	2.01 ± 0.617	n/d	2.72 ± 0.822	n/d	n/d	n/d	0.201 ± 0.0492	n/d	0.0699 ± 0.121

4-Hydroxyphenyllactic Acid	n/d	1.31 ± 0.187	n/d	1.44 ± 0.0699	n/d	2.16 ± 0.227	n/d	1.76 ± 0.301	0.167 ± 0.145	1.82 ± 0.104	0.0581 ± 0.101	1.51 ± 0.229
Phenyllactic Acid	0.0384 ± 0.00258	0.0452 ± 0.00587	0.0397 ± 0.00852	0.0461 ± 0.00725	0.0768 ± 0.00163	0.151 ± 0.133	0.0392 ± 0.00132	0.0474 ± 0.00896	0.145 ± 0.0236	0.0481 ± 0.0278	0.104 ± 0.0130	0.0166 ± 0.00124
Phenylpyruvic Acid	0.109 ± 0.0948	0.238 ± 0.00222	n/d	0.223 ± 0.0193	0.221 ± 0.00538	0.372 ± 0.0431	0.172 ± 0.00794	0.309 ± 0.0246	0.327 ± 0.0515	0.0537 ± 0.0930	0.225 ± 0.0238	0.0489 ± 0.0847
4-Hydroxyphenylpyruvic Acid	0.728 ± 0.104	4.06 ± 0.674	0.518 ± 0.103	3.11 ± 0.445	0.401 ± 0.0575	4.48 ± 0.362	n/d	n/d	n/q ± n/q	n/q ± n/q	n/q ± n/q	n/q ± n/q

**Set E**	Expellers		Seeds		Hemp cake		Hemp fudge		Cream solid residue (wet)		Cream solid residue (dried)	
	Free	Bound	Free	Bound	Free	Bound	Free	Bound	Free	Bound	Free	Bound
Indole	1.72 ± 0.0284	1.75 ± 0.0202	0.885 ± 0.0252	1.03 ± 0.144	1.38 ± 0.0644	1.72 ± 0.271	0.316 ± 0.0278	0.596 ± 0.0834	0.114 ± 0.0161	0.283 ± 0.0156	0.0991 ± 0.00277	0.247 ± 0.0228
Indole-3-Acetic Acid	0.0461 ± 0.0407	0.0943 ± 0.00904	0.0185 ± 0.0321	0.0485 ± 0.0441	0.101 ± 0.00877	0.115 ± 0.00801	0.0833 ± 0.00565	0.0723 ± 0.00474	0.176 ± 0.0193	n/d	0.149 ± 0.0122	n/d

Indole-3-Carboxylic Acid	1.27 ± 0.0771	1.84 ± 0.0571	0.898 ± 0.0131	1.11 ± 0.153	1.16 ± 0.0611	1.53 ± 0.0859	0.347 ± 0.0537	0.391 ± 0.0222	0.261 ± 0.0373	0.353 ± 0.0352	0.222 ± 0.0152	0.275 ± 0.0212
Indole-3-Acrylic Acid	n/d	n/d	n/d	n/d	n/d	n/d	n/d	n/d	n/d	0.00550 ± 0.00953	n/d	0.0123 ± 0.00341
Indole-3-Pyruvic Acid	6.74 ± 1.86	76.6 ± 1.48	1.51 ± 2.62	70.2 ± 8.21	3.22 ± 2.78	83.4 ± 20.3	2.28 ± 2.03	47.3 ± 9.75	n/d	260 ± 11.2	19.4 ± 0.753	170 ± 114
Indoe-3-Lactic Acid	n/d	n/d	n/d	n/d	0.0219 ± 0.0372	n/d	n/d	n/d	n/d	n/d	n/d	n/d
I3-Carboxaldehyde	1.21 ± 0.0501	1.22 ± 0.0473	0.606 ± 0.00646	0.698 ± 0.0961	0.972 ± 0.0594	1.26 ± 0.197	0.205 ± 0.0216	0.472 ± 0.0582	0.123 ± 0.00111	0.323 ± 0.0285	0.0996 ± 0.00616	0.271 ± 0.00834
Niacin	0.955 ± 0.135	8.61 ± 0.301	1.05 ± 0.145	8.23 ± 1.24	2.08 ± 0.178	13.7 ± 1.93	1.69 ± 0.271	12.1 ± 0.726	n/d	0.265 ± 0.0354	n/d	0.215 ± 0.0135
Kynurenic Acid	0.143 ± 0.0174	n/d	0.123 ± 0.0158	n/d	0.181 ± 0.0225	0.0474 ± 0.0821	0.167 ± 0.0118	0.0955 ± 0.00635	0.0798 ± 0.00361	0.258 ± 0.00685	0.0600 ± 0.00982	0.181 ± 0.00596

**Set F**	Expellers		Seeds		Hemp cake		Hemp fudge		Cream solid residue (wet)		Cream solid residue (dried)	
	Free	Bound	Free	Bound	Free	Bound	Free	Bound	Free	Bound	Free	Bound
Ethylferulate	n/d	n/d	n/d	n/d	n/d	0.357 ± 0.619	n/d	n/d	n/d	n/d	n/d	n/d
Chlorogenic Acid	1.41 ± 0.814	n/d	0.412 ± 0.0476	n/d	1.15 ± 0.224	n/d	4.32 ± 1.12	n/d	0.0186 ± 0.0322	n/d	n/d	n/d
Tyrosol	1.95 ± 0.127	9.54 ± 1.84	0.872 ± 0.0872	8.37 ± 0.875	1.75 ± 0.337	11.4 ± 0.898	1.76 ± 0.328	5.88 ± 0.955	0 ± 0	3.78 ± 0.811	n/d	2.32 ± 0.311
Quinadilic Acid	0.0654 ± 0.0118	0.0928 ± 0.00573	0.0594 ± 0.0118	0.0801 ± 0.00733	0.0745 ± 0.0131	0.0944 ± 0.0142	0.0681 ± 0.00483	0.0752 ± 0.00931	0.0576 ± 0.00214	n/d	0.0420 ± 0.00404	n/d
Anthranilic Acid	0.0585 ± 0.00586	1.65 ± 0.0982	0.0607 ± 0.00281	1.36 ± 0.116	0.0729 ± 0.00407	1.84 ± 0.409	0.0678 ± 0.00195	0.968 ± 0.122	0.0472 ± 0.00165	0.572 ± 0.0167	0.0447 ± 0.00573	0.304 ± 0.0242
4-Ethylphenol	n/d	n/d	n/d	n/d	n/d	0.751 ± 0.0172	n/d	0.772 ± 0.0311	n/d	n/d	n/d	n/d

Coniferyl Alcohol	n/d	0.132 ± 0.115	n/d	n/d	0.225 ± 0.0275	0.0674 ± 0.116	0.176 ± 0.00504	n/d	n/d	n/d	n/d	n/d
Catechin	0.107 ± 0.186	n/d	n/d	n/d	0.701 ± 0.103	n/d	0.951 ± 0.124	n/d	n/d	n/d	0.0604 ± 0.104	n/d
Epicatechin	n/d	n/d	n/d	n/d	n/d	n/d	0.503 ± 0.0613	n/d	n/d	n/d	n/d	n/d
Isoliquiritigenin	n/d	n/d	n/d	n/d	n/d	0.0165 ± 0.0287	n/d	n/d	n/d	n/d	n/d	n/d
Naringenin	n/d	n/d	n/d	n/d	n/d	0.0344 ± 0.0596	n/d	n/d	n/d	n/d	n/d	n/d
Naringin	0.197 ± 0.0131	n/d	0.222 ± 0.00319	n/d	0.265 ± 0.00963	n/d	0.192 ± 0.0278	n/d	n/d	n/d	n/d	n/d
Kaempferol	n/d	n/d	n/d	n/d	n/d	0.0891 ± 0.154	n/d	0.101 ± 0.0215	n/d	n/d	n/d	n/d

Morin	n/d	n/d	n/d	n/d	n/d	n/d	n/d	0.0457 ± 0.0792	n/d	n/d	n/d	n/d
Quercetin	n/d	n/d	n/d	n/d	n/d	0.0676 ± 0.117	0.0401 ± 0.0347	0.0954 ± 0.0121	0.0665 ± 0.00368	n/d	0.0411 ± 0.0363	n/d
Myricetin	n/d	n/d	n/d	n/d	n/d	n/d	n/d	n/d	n/d	n/d	n/d	n/d
Quercetin-3-Glucoside	0.396 ± 0.122	0.00245 ± 0.00425	0.302 ± 0.0202	n/d	0.452 ± 0.00897	n/d	0.318 ± 0.0568	n/d	0.0238 ± 0.00341	n/d	0.0150 ± 0.00234	n/d
Taxifolin	0.00672 ± 0.0116	n/d	n/d	n/d	0.0721 ± 0.0112	n/d	n/d	n/d	n/d	n/d	n/d	n/d
Genistein	0.203 ± 0.0226	0.0971 ± 0.0872	0.243 ± 0.00285	0.117 ± 0.00676	0.277 ± 0.00304	0.256 ± 0.173	0.305 ± 0.0344	0.0861 ± 0.0753	n/d	n/d	n/d	n/d
Scopoletin	n/d	n/d	n/d	n/d	n/d	0.00695 ± 0.0121	n/d	n/d	n/d	n/d	n/d	n/d

Quercitrin	0.0383 ± 0.00458	n/d	0.0794 ± 0.0172	n/d	0.0711 ± 0.0214	n/d	0.0638 ± 0.00663	n/d	n/d	n/d	n/d	n/d
Luteolin	0.483 ± 0.0866	1.32 ± 1.51	0.648 ± 0.0987	n/d	0.926 ± 0.145	2.62 ± 2.42	1.44 ± 0.327	0.221 ± 0.194	1.19 ± 0.0472	8.91 ± 4.63	0.893 ± 0.223	3.20 ± 1.24
Formononetin	n/d	n/d	n/d	n/d	n/d	n/d	n/d	n/d	n/d	n/d	n/d	n/d
Apigenin	0.168 ± 0.0183	0.147 ± 0.0696	0.207 ± 0.00354	0.133 ± 0.0497	0.236 ± 0.00532	0.243 ± 0.117	0.257 ± 0.0303	0.197 ± 0.0149	0.107 ± 0.0114	0.152 ± 0.104	0.0898 ± 0.00686	0.0799 ± 0.0178
Luteolinidin	n/d	n/d	n/d	0.0121 ± 0.0224	n/d	n/d	n/d	n/d	n/d	n/d	n/d	n/d
Coumesterol	n/d	0.0121 ± 0.0208	0.0121 ± 0.0211	0.0127 ± 0.0223	n/d	n/d	n/d	n/d	n/d	n/d	n/d	n/d
8-Methylpsoralen	0.00298 ± 0.00516	n/d	n/d	n/d	n/d	n/d	n/d	n/d	n/d	n/d	n/d	n/d

Bergapten	n/d	n/d	n/d	n/d	n/d	n/d	n/d	n/d	n/d	n/d	n/d	n/d
Tangeretin	0.0166 ± 0.00436	0.0283 ± 0.000697	0.0146 ± 0.000948	0.0287 ± 0.00257	0.00996 ± 0.00865	0.0261 ± 0.00818	0.0138 ± 0.000451	0.0327 ± 0.00236	n/d	n/d	n/d	n/d
Imperatorin	0.0119 ± 0.0103	n/d	0.0121 ± 0.0104	0.00711 ± 0.0122	0.0118 ± 0.0102	n/d	0.0118 ± 0.0102	n/d	n/d	n/d	n/d	n/d
Rutin	0.0528 ± 0.00484	n/d	0.0372 ± 0.0324	n/d	0.0777 ± 0.00117	n/d	0.116 ± 0.0211	n/d	n/d	n/d	n/d	n/d
Isorhamnetin	n/d	n/d	n/d	n/d	n/d	1.52 ± 2.52	n/d	n/d	n/d	0.0851 ± 0.00591	n/d	0.0497 ± 0.0431
Vitexin	0.251 ± 0.0383	2.06 ± 0.0228	0.271 ± 0.0142	1.95 ± 0.227	0.362 ± 0.0179	2.72 ± 0.477	0.603 ± 0.0971	3.11 ± 0.437	0.0321 ± 0.00108	0.321 ± 0.0676	0.0296 ± 0.00135	0.238 ± 0.0401
Hyperoside	0.406 ± 0.129	0.00597 ± 0.0103	0.306 ± 0.0305	0.00509 ± 0.00445	0.457 ± 0.0222	n/d	0.324 ± 0.0566	n/d	0.0125 ± 0.00353	n/d	0.00738 ± 0.00186	n/d

Glycitein	0.0202 ± 0.0175	n/d	0.0121 ± 0.0173	n/d	0.0305 ± 0.00152	n/d	0.0361 ± 0.00154	n/d	0.0113 ± 0.0196	n/d	0.0102 ± 0.0177	n/d
**Set G**	Expellers		Seeds		Hemp cake		Hemp fudge		Cream solid residue (wet)		Cream solid residue (dried)	
	Free	Bound	Free	Bound	Free	Bound	Free	Bound	Free	Bound	Free	Bound
Secoisolariciresinol	n/d	1.15 ± 0.119	n/d	0.693 ± 0.144	n/d	1.27 ± 0.213	n/d	0.206 ± 0.0201	n/d	n/d	n/d	n/d
Syringaresinol	n/d	116 ± 9.48	n/d	73.2 ± 4.26	n/d	125 ± 7.53	n/d	15.6 ± 4.17	n/d	0.351 ± 0.609	n/d	n/d
Pinoresinol	0.117 ± 0.0145	1.11 ± 0.0629	0.0728 ± 0.00446	0.629 ± 0.0442	0.172 ± 0.00953	1.25 ± 0.0475	0.0856 ± 0.0111	0.117 ± 0.0527	n/d	0.00791 ± 0.00681	n/d	0.00697 ± 0.00815

Where n/d = not detected (i.e., below the detection level).

Table S3. Percentage of recommended dietary allowances of fatty acid methyl esters met by the hemp seed-based foods and the by-products in a consumption of 100g ± SD (n = 3)

Sample type	Percentage of dietary allowances for fatty acid methyl esters measured from 100g of hemp seed-based samples				
	**Palmitic acid**	**Stearic acid**	**Linoleic acid**	**Gamma-linolenic acid**	**Alpha- linolenic acid**
**Protein fibre boost**	29.9 ± 0.0497	43.7 ± 0.0306	597 ± 0.0917	30.6 ± 0.00463	790 ± 0.0221
**Protein-75-product**	24.6 ± 0.0999	47.9 ± 0.0724	578 ± 1.31	26.3 ± 0.153	815 ± 0.466
**Protein-85-product**	34.6 ± 1.65	61.1 ± 0.175	538 ± 2.02	22.3 ± 0.153	600 ± 0.666
**Protein-46-product**	30.2 ± 0.107	51.9 ± 0.0328	593 ± 0.0573	26.3 ± 0.0154	665 ± 0.131
**Hemp seed-hull flour**	30.1 ± 0.127	45.7 ± 0.0705	601 ± 0.0317	27.1 ± 0.00938	715 ± 0.192
**Hemp seed hearts**	23.6 ± 0.0614	43.7 ± 0.0339	593 ± 0.0872	27.1 ± 0.00803	790 ± 0.0308
**Expellers**	29.5 ± 0.411	48.2 ± 0.0188	592 ± 0.622	26.5 ± 0.0531	705 ± 0.153
**Seeds**	24.8 ± 0.0265	44.3 ± 0.0361	611 ± 0.101	27.4 ± 0.0121	805 ± 0.0532
**Hemp cake**	30.3 ± 0.188	50.5 ± 0.0929	607 ± 0.362	26.2 ± 0.0309	740 ± 0.239
**Hemp fudge**	23.7 ± 0.0727	42.8 ± 0.0861	585 ± 0.309	27.1 ± 0.0223	755 ± 0.108
**Cream solid residue (wet)**	26.5 ± 0.0935	47.1 ± 0.0291	555 ± 0.375	29.1 ± 0.0106	715 ± 0.0436
**Cream solid residue (dried)**	26.1 ± 0.182	41.8 ± 0.171	564 ± 0.171	25.7 ± 0.0667	730 ± 0.0932
**Hemp oil**	19.7 ± 0.361	38.4 ± 0.0288	551 ± 0.546	30.5 ± 0.209	810 ± 0.252
**Hemp cream**	25.7 ± 0.0733	49.6 ± 0.0443	695 ± 0.452	32.6 ± 0.0202	940 ± 0.142

Recommended dietary allowances in g/100g: **palmitic acid**, 30; **stearic acid**, 6.95; **linoleic acid**, 10; **gamma-linolenic acid**, 10; **alpha-linolenic acid**, 2.

There is not published data available for the recommended dietary allowance of oleic acid.

Table S4. Percentage of the reference nutrient intake for micronutrient content met by the hemp seed-based foods and the by-products in a consumption of 100 g ± SD (n = 3)

**Sample type**	**Percentage of the reference nutrient intake measured from 100g of intake of hemp seed-based samples**								
	**Na**	**K**	**Ca**	**Mg**	**P**	**Mn**	**Fe**	**Cu**	**Zn**
**Protein fibre boost**	0.00553 ± 0.00678	33.3 ± 0.486	53.4 ± 0.841	209 ± 2.82	240 ± 2.66	833 ± 8.56	272 ± 4.57	144 ± 1.31	85.6 ± 0.844
**Protein-75-product**	1.08 ± 0.0108	28.1 ± 0.431	40.1 ± 0.442	527 ± 1.91	573 ± 21.3	1056 ± 8.98	416 ± 2.14	82.7 ± 1.28	290 ± 3.03
**Protein-85-product**	0.448 ± 0.00304	3.85 ± 0.0521	23.8 ± 0.461	133 ± 2.47	355 ± 5.25	575 ± 33.2	398 ± 6.87	68.1 ± 2.33	260 ± 3.86
**Protein-46-product**	0 ± 0	40.8 ± 1.41	40.3 ± 1.58	272 ± 9.35	322 ± 10.1	832 ± 16.4	256 ± 4.96	146 ± 3.21	133 ± 3.83
**Hemp seed-hull flour**	0 ± 0	13.1 ± 1.03	28.8 ± 2.76	47.8 ± 6.14	40.7 ± 3.67	408 ± 39.4	120 ± 17.2	76.1 ± 6.22	18.0 ± 2.29
**Hemp seed hearts**	0.0356 ± 0.0616	24.8 ± 0.563	15.4 ± 0.417	169 ± 3.19	200 ± 6.94	328 ± 6.83	123 ± 2.04	93.5 ± 3.27	88.4 ± 2.16
**Expellers**	0 ± 0	21.6 ± 0.903	29.7 ± 1.61	110 ± 6.63	118 ± 5.33	473 ± 22.5	117 ± 7.15	108 ± 4.71	50.4 ± 3.61

**Seeds**	0 ± 0	19.3 ± 0.384	24.1 ± 1.42	116 ± 3.43	128 ± 2.54	407 ± 20.6	105 ± 3.11	89.3 ± 3.82	55.1 ± 2.61
**Hemp cake**	0.00561 ± 0.00987	26.1 ± 0.639	33.2 ± 0.896	150 ± 7.44	164 ± 2.28	584 ± 38.5	146 ± 6.54	124 ± 5.62	72.6 ± 3.97
**Hemp fudge**	0 ± 0	28.1 ± 3.05	38.4 ± 4.03	202 ± 19.6	234 ± 25.1	388 ± 38.8	179 ± 16.7	117 ± 11.4	97.2 ± 9.84
**Cream solid residue (wet)**	0.471 ± 0.0102	34.1 ± 0.144	20.2 ± 0.434	173 ± 1.43	224 ± 2.71	384 ± 3.73	166 ± 1.63	132 ± 1.48	115 ± 1.71
**Cream solid residue (dried)**	0.415 ± 0.0294	31.8 ± 2.77	22.8 ± 1.81	187 ± 14.8	239 ± 21.1	440 ± 38.7	188 ± 15.4	133 ± 9.61	127 ± 11.1
**Hemp oil**	0.0142 ± 0.0114	0.0227 ± 0.00542	0.232 ± 0.122	0.0255 ± 0.00452	0 ± 0	0.655 ± 0.0647	0.912 ± 0.185	0.451 ± 0.169	0.291 ± 0.502
**Hemp cream**	0.0510 ± 0.0102	3.01 ± 0.232	0.343 ± 0.0111	2.74 ± 0.159	8.33 ± 0.721	2.31 ± 0.405	2.44 ± 0.111	13.7 ± 0.825	0.423 ± 0.312

The microelements reference nutrient intake (RNI) in mg/100g: **sodium (na)**, 1600; **potassium (k)**, 3500; **calcium (ca)**, 700; **magnesium (mg)**, 300; **phosphorus (p)**, 540; **manganese (mn)**, 2.30; **iron (fe)**, 8.70; **copper (cu)** 1.20; **zinc (zn)**, 9.50.
